# Web-Based Risk Communication and Planning in an Obese Population: Exploratory Study

**DOI:** 10.2196/jmir.1579

**Published:** 2011-11-24

**Authors:** Anastasia Soureti, Peter Murray, Mark Cobain, Willem van Mechelen, Robert Hurling

**Affiliations:** ^1^Unilever DiscoverColworth Science ParkBedfordshireUnited Kingdom; ^2^Department of Public and Occupational Health and EMGO Institute for Health & Care ResearchVU University Medical CentreAmsterdamNetherlands

**Keywords:** Risk perceptions, cardiovascular disease, planning, saturated fat intake

## Abstract

**Background:**

A healthy diet, low in saturated fat and high in fiber, is a popular medical recommendation in preventing cardiovascular disease (CVD). One approach to motivating healthier eating is to raise individuals’ awareness of their CVD risk and then help them form specific plans to change.

**Objectives:**

The aim was to explore the combined impact of a Web-based CVD risk message and a fully automated planning tool on risk perceptions, intentions, and saturated fat intake changes over 4 weeks.

**Methods:**

Of the 1187 men and women recruited online, 781 were randomly allocated to one of four conditions: a CVD risk message, the same CVD risk message paired with planning, planning on its own, and a control group. All outcome measures were assessed by online self-reports. Generalized linear modeling was used to analyze the data.

**Results:**

Self-perceived consumption of low saturated fat foods (odds ratio 11.40, 95% CI 1.86–69.68) and intentions to change diet (odds ratio 21.20, 95% CI 2.6–172.4) increased more in participants allocated to the planning than the control group. No difference was observed between the four conditions with regard to percentage saturated fat intake changes. Contrary to our expectations, there was no difference in perceived and percentage saturated fat intake change between the CVD risk message plus planning group and the control group. Risk perceptions among those receiving the CVD risk message changed to be more in line with their age (change in slope_individual_ = 0.075, *P* = .01; change in slope_comparative_ = 0.100, *P* = .001), whereas there was no change among those who did not receive the CVD risk message.

**Conclusion:**

There was no evidence that combining a CVD risk message with a planning tool reduces saturated fat intake more than either alone. Further research is required to identify ways in which matching motivational and volitional strategies can lead to greater behavior changes.

**Trial Registration:**

International Standard Randomized Controlled Trial Number (ISRCTN): 91154001; http://www.controlled-trials.com/ISRCTN91154001 (Archived by WebCite at http://www.webcitation.org/62sBoGeOO)

## Introduction

Cardiovascular disease (CVD) is a leading cause of death among adults [1]. A healthy diet, low in saturated fat and high in fiber, is a popular medical recommendation in preventing CVD.

One approach to increase motivation to change is to improve awareness of the risk associated with an unhealthy lifestyle [2,3]. Risk analogies such as Heart-Age (HA) combine aspects of absolute and relative CVD risk and have been found effective in communicating future CVD risk [4,5]. In a recent study, those at higher actual CVD risk who received a HA risk analogy were more aware of their future CVD risk than were those exposed to a percentage CVD risk score [4].

Although many people report having good intentions to eat more healthily, these are not always translated into action [6,7]. Action plans, also known as implementation intentions, are strategies that can bridge the gap between intention and behavior. A meta-analysis of 94 studies showed that implementation intentions had a medium to large effect on goal achievement [8]. Fear appeals may also facilitate change when they are combined with specific instructions on what action to take [9]. While earlier studies explored the value of using action plans [7,10], more recently there has been a greater interest in the characteristics and mechanisms underlying effective plans [11-18], such as the creation of a strong cue–response relationship [12,13].

Research has also investigated the impact of self-efficacy on behavior change. According to the health action process approach model, *action* self-efficacy acts on the motivational part of decision making, whereas *maintenance* self-efficacy acts on the volitional part of the behavior [3]. While some studies report higher self-efficacy in participants making an implementation intention [19,20], others find no difference [7].

Implementation intention research to date has been largely offline (paper and pencil) with little focus and mixed results when their effectiveness has been tested online [21,22]. In a study conducted in an occupational setting, use of online implementation intentions backfired, such that participants who did not form an implementation intention exercised significantly more than participants who formed an implementation intention [21]. In an online dietary intervention, implementation intentions were combined with a text message reminder service leading to a reduction in perceived saturated fat intake and portion sizes [22]. The present study is one of a few studies designed to act on both the motivational and volitional phase of behavior change [23]. We offered a risk communication message to create more appropriate risk perceptions and to increase intention to change, and then helped individuals change their dietary behavior by forming specific plans on how to achieve this. This is also one of the few studies that compared the independent and combined short-term effects of an online health risk communication message and an online implementation intention tool on the promotion of healthy eating in an obese population, who are more likely to be at risk of developing CVD.

### Objectives

The primary aim of this investigatory study was to test whether participants could form plans via a fully automated Web-based planning tool (PT) and to assess the short-term effects of combining a CVD risk message (Heart-Age, HA) with the planning tool (HA+PT) on participants’ saturated fat intake, measured by a 2-item scale (TIS) and a food frequency questionnaire (FFQ) over a period of 3 weeks. A secondary aim was to assess the effects of the heart-age risk message and planning tool on participants’ risk perceptions, self-efficacy, intentions to change saturated fat intake, and intentions to test cholesterol and blood pressure levels. We expected that the heart-age message would primarily change risk perceptions and participants’ intentions to change, while the planning tool would act primarily on self-efficacy and behavior. We wanted to explore whether participants could form Web-based plans and whether the combined HA+PT intervention would have a greater impact than either the heart-age message or the planning tool alone.

## Methods

### Participants

We invited 1187 participants through an online recruitment agency to log in to an open access website to take part in the study. The self-report eligibility criteria included age (30–60 years), obesity (body mass index [BMI] ≥29 kg/m^2^), not having a diagnosis of a heart condition or cancer or being pregnant, and being computer savvy. We chose obese participants because they were likely to benefit from heart-health information [24]. To help minimize any imbalance effects created by smokers receiving a higher heart-age score, a UK-representative sample of smokers was distributed across the four conditions of the study.

### Design and Procedures

This study was conducted between the middle of January and the end of February 2009 and has been registered retrospectively. It was a Web-based, randomized, between-groups study designed to assess the difference in saturated fat intake between four experimental conditions. No participant–experimenter contact was present. Participants were given online instructions and completed each week’s session from the convenience of their home computer. At week 1 (recruitment), participants were recruited by an online agency, signed an online consent form [25], and completed an online questionnaire on their current saturated fat intake, risk perceptions, self-efficacy, and intentions to change their dietary intake. They also received educational information on the importance of a healthy diet low in saturated fat (Multimedia Appendix 1).

At week 2 (intervention), those participants who returned to the website were randomly allocated, using a computer-generated list of random numbers, into one of four conditions: (1) control group (CG), (2) PT condition, (3) HA risk message condition, and (4) HA+PT condition. Allocation of the participants in the four conditions was also stratified to balance by age group (30–45 years or 46–60 years) and gender. In the groups that received the HA risk message, participants filled out online information on their age, gender, weight, height, prescribed blood pressure medication, family history of heart and vascular disease, smoking status, self-prevalent diabetes, self-reported total and high-density lipoprotein cholesterol levels, and systolic blood pressure. They then received feedback on their future CVD risk in the form of the HA risk message. Participants in the PT condition were asked to identify a list of situations in which they would like to change their saturated fat intake and match these situations with a list of behaviors. Participants in all conditions were asked to fill out a shorter version of the questionnaire asked at baseline. At week 2, participants completed the session once and were not able to revisit the website to make any changes (eg, to create more plans).

At week 5 (follow-up), participants were asked to complete a follow-up assessment. They received £15 on study completion and were entered in a prize draw for vouchers (£200).

### Interventions

#### The Heart-Age Risk Message Condition

**Figure 1:**
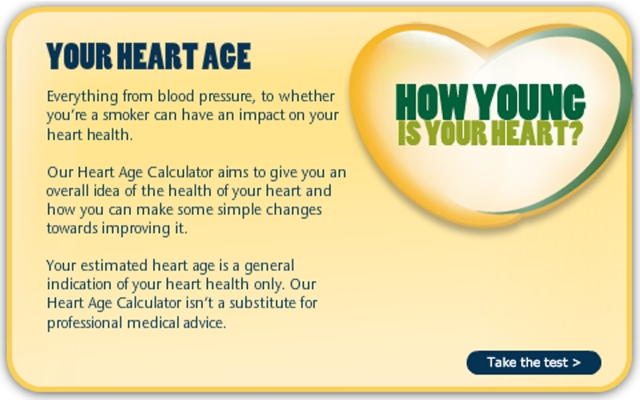
The Heart-Age risk message.

Heart-age, which is described in more detail elsewhere [26], is the age corresponding to someone of the same gender with the same CVD risk level but with normal risk factors. The definition of normal is based on the following profile: not smoking, not diabetic, systolic blood pressure 125 mmHg (midpoint of normal range: 120–130 mmHg), total serum cholesterol 180 mg/dL (4.66 mmol/L; between normal range of 160–200 mg/dL or 4.14–5.18 mmol/L), and high-density lipoprotein cholesterol 45 mg/dL (1.17 mmol/L). For example, a 61-year-old man who smokes and has no other risk factors has a 10-year CVD risk of 10% and the HA of a 73-year-old man. In the HA condition, users filled in an online questionnaire and received feedback in form of the HA risk message (Figure 1).

#### The Planning Tool Condition

Participants who received the PT selected from a list of 13 situations, in which they were tempted to eat unhealthily and then chose an approach to change their behavior from a list of 13 solutions. For every situation–solution pair chosen, a line was drawn visually linking the two together [27]. Participants were asked to complete at least 3 situation–solution pairs.

**Figure 2:**
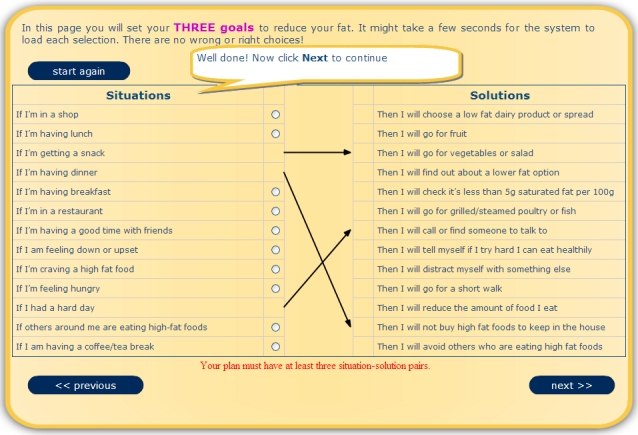
The planning tool.

The solutions were based on constructs from the processes of change model (eg, counterconditioning, stimulus control, and helpful relationships) [28]. Some nutritionally based behaviors were also included from an accredited site [29] after review by an expert nutritionist. The list of situations consisted of both situational cues (eg, having lunch) focusing on the “when and where” and motivational cues (eg, feeling bored) linked to the reasons (“why”) for performing a specific behavior [30]. Motivational cues were divided into three main situations: (1) experiencing positive affect, (2) experiencing negative affect, and (3) being faced with cravings [31,32]. The situations were translated into “if” statements (eg, “If I’m having breakfast”) and the list of solutions was translated into “then” statements (eg, “then I will tell myself I can eat healthily”). Figure 2 shows the PT.

#### Control Group Condition

Participants in the CG received educational information on the importance of a healthy diet low in saturated at week 1 and filled out the same online questionnaires as the rest of the experimental conditions at all study weeks. 

### Outcome Measures

*Saturated fat intake,* as the primary outcome measure, was assessed at baseline and follow-up by two measures. First, a self-report index of food [33] was used to record the frequency of consumption of 63 common foods. This FFQ has good test–retest reliability (*r* = .62, *P* < .01) [33] and validity when compared with 10-day weighed records [34,35]. Second, a two item scale (TIS) (*r* = .78, *P* < .001) was adapted from a previous study [34]. Participants were asked to report their agreement in consumption of low saturated fat foods (“I have eaten foods low in saturated fat...”) followed by frequency in consumption of these foods (“How often did you eat foods low in saturated fat?”). The correlation between the two measures was –0.320 (*P* < .001) at week 1 and –0.291 (*P* < .001) at week 5. Negative correlations are due to reverse scales used for the self-perceived items.

*CVD risk perceptions* measured participants’ perceived risk in an absolute sense and comparative with their age group [4]. The first item (Q1) examined perceptions of individual CVD risk (“I think that my chances of getting heart disease in the short term are...”). The second item (Q2) compared participants’ risk perceptions against those of other people of their age (“Compared to an average person of my age and sex, my chances of getting heart disease are...”). Responses were measured on a 7-point Likert scale at weeks 1 and 2.

*Intention* to reduce saturated fat intake was measured at weeks 1, 2, and 5 on a 7-point Likert scale via 10 items, which were highly intercorrelated (Cronbach alpha = .92), so were analyzed as a composite score. At follow-up, there were two further questions on participants’ intentions to assess their cholesterol and blood pressure over the next month.

*Action and maintenance self-efficacy* were modified from previous research [3,36-38]. Action self-efficacy (alpha = .84), which was measured at all study times, consisted of 4 items focusing on confidence to overcome obstacles. Maintenance self-efficacy (alpha = .89), assessed only at follow-up, consisted of 11 items exploring confidence in sustaining change in the face of difficulties. Items were measured on a 4-point scale (not at all, barely true, mostly true, exactly true).

*Planning and outcome expectancies* items were adapted from previous research [3,36-38] and measured on a 4-point scale. Planning comprised 2 items: “I have my own plan regarding (1) when, (2) how to reduce my saturated fat intake.” Outcome expectancies consisted of 11 items linked to the positive and negative expectancies of reducing saturated fat intake (eg, “If I reduce my saturated fat intake”... “food won’t taste as good,” “I will feel good”).

*Feedback on the intervention* was assessed at week 2 and at follow-up on a 7-point Likert scale (ranging from strongly disagree to strongly agree). Participants were asked to rate the intervention in terms of its emotional impact, personal relevance, interest, trustworthiness, credibility, and enjoyment. All items were adapted from previous studies [39-41].

### Statistical Considerations

Analysis of the outcome measures was restricted to those respondents who completed the follow-up assessments. Response to the CVD risk perceptions was analyzed using a generalized linear model with a cumulative logistic link function and multinomial distribution. Baseline scores and heart risk-adjusted age were included as covariates. As with all the analyses other potential covariates (eg, smoking, BMI, social economic status) were retained if significant in the model. Similar models were used for the intention-to-change and intention-to-test questions, and self-efficacy, planning, and feedback items, but omitting the heart risk-adjusted age covariate.

Mean change in self-perceived saturated fat intake within a group was assessed using analysis of variance with baseline included as a covariate. The groups were compared using another generalized linear model with a cumulative logistic link function and multinomial distribution. Data from the index of food was summarized to yield the total calorie intake per participant and the percentage of total energy intake contributed by saturated fat. We analyzed all of these data using analysis of variance models with baseline covariates always included and any other significant covariates retained. All analysis was carried out using version 9.1.3 of SAS software (SAS Institute, Cary, NC, USA).

### Local Research Ethical Review Requirement

The study protocol (Multimedia Appendix 1) was approved by an independent research ethics committee (Colworth Research Ethics Committee) in the South of England on December 4, 2008 (Multimedia Appendix 2). All research was conducted in accordance with the Declaration of Helsinki [42].

## Results

### Participant Baseline Characteristics

**Figure 3:**
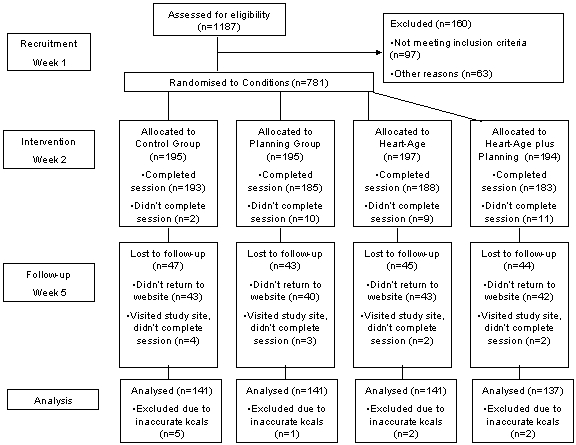
Flow chart of recruitment, intervention, and follow-up.

At week 1, we invited 1187 people to participate through an online recruitment agency, of whom 1027 completed the initial questionnaire and were invited to take part in the study. At week 2, a total of 781 participants revisited the website and were allocated to one of four conditions. At week 2, 32 of these participants did not complete the online session. At week 5, a total of 581 participants returned to complete the follow-up questionnaire. We excluded 21 participants from the statistical analysis because they did not complete the whole session or due to inaccurate calorie intake reporting (<500 kcal or >5000 kcal per day). The numbers of participants completing each week are shown in Figure 3.

There was no significant difference in percentage saturated fat intake between participants who completed only the week 1 assessment and those who completed the week 5 measures (*P* = .79). The mean percentage saturated fat intake at week 1 (baseline) was 15.4%, much higher than the UK recommended levels [43]. Table 1 shows participants’ baseline characteristics.

**Table 1:** Participants’ baseline characteristics

	Overall	Control group	Planning group	Heart-Age group	Planning plus Heart-Age group	*F*_3,777_ statistic (*P* value)
Number	781	195	195	197	194	
Age (years)^a^, mean (SD)	46.89 (8.26)	47.05 (8.48)	47.06 (8.11)	46.91 (8.00)	46.56 (8.52)	0.15 (.93)
BMI^b^ (kg/m^2^)^a^, mean (SD)	35.71 (5.71)	35.72 (5.40)	35.51 (6.15)	36.49 (6.42)	35.08 (4.64)	2.10 (.1)
Smokers (%)	25.61	25.13	25.64	25.26	26.40	0.10^c^ (.99)

^a^ No significant differences found in participants’ baseline characteristics (*P* > .05).

^b^ Body mass index.

^c^ Chi-square test (c^2^_3_) statistic and *P* value.

### Planning Tool

All participants allocated to the PT condition were able to formulate their Web-based plans with an average of 3.9 plans. Participants selected a range of motivational and situational cues. The most frequently chosen situations were “If I’m feeling hungry” (99/747, 13%), “If I’m getting a snack” (97/747, 13%), “If I’m having lunch” (71/747, 10%) “or dinner” (74/747, 10%), “If I’m craving a high-fat food” (66/747, 9%), and “If I’m feeling down or upset” (72/747, 10%).

The most frequently selected solutions were “Then I will go for fruit” (149/747, 19.7%), “Then I will find out about a lower-fat option” (105/747, 14.1%), “Then I will go for grilled/steamed poultry or fish” (85/747, 11%), “Then I will distract myself with something else” (70/747, 9%), and “Then I will tell myself if I try hard I can eat healthily” (66/747, 9%).

#### Time Spent Online

At week 1, participants spent an average of 12.44 (SD 9.77) minutes online. At week 2, the CG spent the least time online (mean 4.19, SD 2.43 minutes), followed by the PT (mean 7.84, SD 5.18 minutes), the HA (mean 10.91, SD 8.46 minutes), and lastly the HA+PT group (mean 12.47, SD 6.48 minutes). HA+PT spent significantly more time online than the PT group (95% CI, 2.73–6.53) or the CG (95% CI, 6.39–10.18). No significant differences were found at week 2 between the HA+PT and the HA-only condition (95% CI, –0.34 to 3.46). At week 5, there were no further significant differences (*P* = .67) between the four conditions in time spent filling out the follow-up questionnaire (CG: mean 9.54, SD 3.62 minutes; PT: mean 11.38, SD 9.84 minutes, HA: mean 10.44, SD 5.47 minutes; HA+PT: mean 9.64, SD 6.32 minutes).

### Primary Outcomes

#### Saturated Fat Intake

Participants in all four conditions reported a significant increase in consumption of foods low in saturated fat (the mean of the two self-perceived intake items) between baseline and follow-up, apart from the CG (Table 2). The generalized linear model analysis showed a significant difference between the conditions (c^2^_3_ = 13.1, *P* = .005) with respect to perceived saturated fat intake changes. Multiple comparisons of the conditions (with Bonferroni adjustment to allow for the six comparisons) indicated this was due to participants in the PT group reporting a higher perceived increase in low saturated fat foods than those in the CG (odds ratio, 11.40; 95% CI, 1.86–69.68).

**Table 2:** Saturated fat intake by primary outcome (self-perceived and index of food questionnaire)

Condition	Self-perceived items (baseline mean 4.73)			Index of food questionnaire (baseline mean 15.37%)		
	Week 5	Week 5 – week 1^a^	Pr > |t|	Week 5	Week 5 – week 1^a^	Pr > |t|
Control group	4.857	0.125 (0.100)	.21	14.67	–0.717 (0.198)	.0003
Planning tool	5.087	0.355 (0.102)	.001	14.51	–0.875 (0.198)	<.0001
Heart-Age	4.943	0.212 (0.101)	.04	14.63	–0.748 (0.197)	.0002
Heart-Age + planning tool	4.977	0.245 (0.102)	.02	14.49	–0.893 (0.200)	<.0001

^a^ Mean and standard error after adjusting for baseline and other covariates. Note that the standard error for week 5 is the same as the standard error for weeks 5 – 1, due to the use of a baseline covariate in the analysis.

With regard to the index of food, participants in all conditions reported a significant reduction in percentage saturated fat intake between baseline and follow-up (Table 2 with no significant differences found between the four conditions (*P* = .89).

### Secondary Outcomes

#### Risk Perceptions

**Figure 4:**
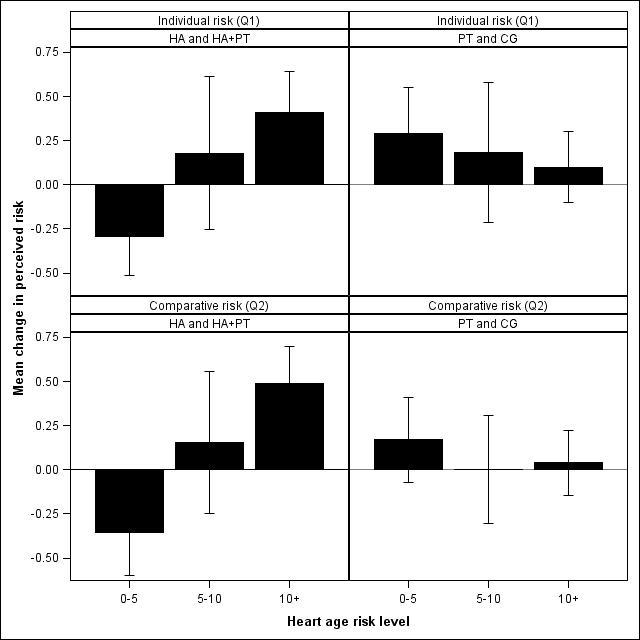
Change in risk perceptions for Q1 and 2 split by Heart-Age level.

The generalized linear model found no significant differences between the four experimental conditions in terms of their CVD risk perceptions, both for individual (Q1) (*P* = .88) and comparative risk (Q2) (*P* = .93). In order to test whether perceived risk was more related to actual risk, we further compared the change in perceived risk between week 2 and week 1 for all participants who received the HA risk message (HA, HA+PT) with those who did not (PT, CG) using a further generalized linear model. Figure 4 shows risk perception changes for Q1 and Q2 split by the different HA risk levels (low: 0–5, moderate: 5–10, and high: 10–15). HA level is the difference between an individual’s actual age and his or her risk-adjusted age. For example, the 0–5 HA level includes people whose HA is up to 5 years older than their actual age. For both Q1 and Q2, the regression slopes for those in the HA conditions moved to be more in line with participants’ HA risk levels, whereas this was not found for those in the non-HA conditions.

Specifically, for participants in the HA risk message conditions, there was a significant increase in the regression slope of the individual risk perception question (Q1) against the risk-adjusted age (change in slope 0.075, SE 0.029, *P* = .01) after participants were shown the risk message. There was no significant change (change in slope –0.027, SE 0.031, *P* = .38) for those participants in the non-HA risk message conditions. A similar pattern was found for the comparative risk question (Q2), with a statistically significant change in the regression slope against the risk-adjusted age for the HA risk message groups (change in slope 0.100, SE 0.030, *P* = .001), but no significant change for the non-HA risk message groups (change in slope –0.029, SE 0.030, *P* = .34).

#### Intentions to Change

Generalized linear modeling showed that change in intention to reduce saturated fat intake at week 2 compared with week 1 was significantly influenced by condition (c^2^_3_ = 18.8, *P* < .001). Multiple comparisons between conditions (with a Bonferroni adjustment to allow for the six comparisons) showed that participants in the PT condition had a much higher intention than those allocated to the CG (odds ratio, 21.20; 95% CI, 2.6–172.4) or the HA risk message condition (odds ratio, 0.04; 95% CI, 0.0054–0.42).

There were no significant differences between the conditions for intention to take a cholesterol (*P* = .38) or blood pressure test within the next month (*P* = .90). There was a significant gender-by-group interaction (c^2^_3_ = 13.6, *P* = .004). Comparisons within the interaction effect (with a Bonferroni multiplicity adjustment) indicated that women who received the HA risk message were more motivated than the women in the CG to get their cholesterol tested within the next month (odds ratio, 2.46; 95% CI, 1.14–5.28). The same was true for women when the HA+PT condition was compared against the CG (odds ratio, 2.60; 95% CI, 1.18–5.76). There was no significant effect of condition on intention to test blood pressure (c^2^_3_ = 0.8, *P* = .85) and no interaction with gender.

#### Self-Efficacy

The generalized linear model showed that action self-efficacy measured at week 2 differed significantly between the conditions (c^2^_3_ = 16.6, *P* < .001). This was due to participants in the PT group being more confident than those in the CG (odds ratio, 3.06; 95% CI, 1.40–6.66). This difference was not statistically significant at week 5 (c^2^_3_ = 7.1, *P* = .07). Maintenance self-efficacy measured at week 5 was not significantly different between the four conditions (*P* = .45).

#### Planning and Outcome Expectancies

At week 5, there was no significant difference in the “how” (*P* = .87) or “when” (*P* = .60) to reduce saturated fat intake between the four conditions. There were no significant effects of conditions for any of the outcome expectancy items.

#### Feedback on the Intervention

At week 2, there was a significant difference in perceived trustworthiness (c^2^_3_ = 8.9, *P* = .03), with those receiving the HA+PT reporting the intervention to be less trustworthy than those receiving the PT alone (mean 5.6 vs 5.9). There was also a difference between conditions for “informative” (c^2^_3_ = 14.3, *P* = .003) with HA+PT being perceived as less informative than the HA alone or the CG (mean 5.8 vs 6.1 vs 6.04). There was an overall difference in “worried” scores (c^2^_3_ = 4.8, *P* = .03). The HA+PT (mean 4.6) and the HA risk message participants (mean 4.7) were more worried than the PT participants (mean 4.0). All other feedback items were not significant. At week 5, there was still a significant difference between the conditions for “interesting” (c^2^_3_ = 8.6, *P* = .04), with the HA+PT participants still reporting the experience as less interesting than those receiving HA alone (mean 5.4 vs 5.7).

## Discussion

### Principal Results

In this study, a fully automated planning tool was successfully used by participants to form a set of health plans. The planning tool boosted self-efficacy and intention and reduced perceived saturated fat intake for one of the measures (TIS) but not the other (FFQ). A CVD risk message improved people’s awareness of their risk relative to their age. Contrary to our expectations, combining a CVD risk message with the planning tool did not lead to bigger reductions in saturated fat intake than when they were presented on their own.

In line with theories of behavior change [36-38], the planning tool was better than the control group at increasing self-perceived consumption of low saturated fat foods (TIS). The same finding was not true for our second measure of saturated fat intake (FFQ). Also, participants in all conditions reported a change in percentage saturated fat intake measured by the FFQ, whereas participants in all conditions apart from the control group reported a change in their TIS score. Similar findings in terms of discrepancies between the FFQ and self-perceived items have been reported before [17]. This implies that the two self-perceived saturated fat intake items were better able than the FFQ measure to differentiate between the conditions. However, both come with limitations, which we discuss in the next section.

In line with our hypothesis, the planning tool was also better than the control group at boosting participants’ intentions to reduce saturated fat [18] and action self-efficacy in the short term [19,20]. However, maintenance self-efficacy did not differ between the conditions at follow-up. This might be because participants who formed plans and encountered difficulties needed further support (eg, coping plans) to maintain their healthy eating. A previous study found that action plans are more effective at the early stages of change, while coping plans are instrumental at later stages [37].

In support of previous studies, receiving the heart-age risk message led to more appropriate risk perceptions [4,5,44], linked to participants’ risk relative to their age group. Presentation of risk information also increased women’s intentions to test their cholesterol. The latter finding is important because people who are aware of their cholesterol levels can receive more precise risk estimates.

Contrary to our expectations, combining the heart-age risk message with the planning tool (HA+PT) did not lead to a bigger reduction in saturated fat intake. A mismatch might have been created between the global CVD risk message and the specific target plan, confusing smokers with a high heart-age, who saw smoking cessation as the primary route to better health rather than diet. Alternatively, cognitive overload might have confounded the impact of HA+PT on saturated fat intake [45,46]. The length of time spent interacting online may also have been a factor, with the HA+PT taking the longest (12.47 minutes vs 10.91 for HA and 7.84 for PT). Future research could explore whether there is a benefit from reducing cognitive load through the use of a delay between presenting risk information and forming plans.

### Limitations, Advantages, and Future Studies

The impact of conditions on our two measures of saturated fat intake changes was inconsistent, and this could be due to the limitations present in the FFQ and the TIS. Underreporting of food consumption is a recurrent challenge for FFQs and is most pronounced among overweight and obese people [47]. Also, FFQs were initially designed to estimate individual intake relative to a population rather than to detect small changes in individual dietary intake [33,48], for which they might not be sufficiently sensitive. The present FFQ did not account for individual variation in portion sizes but instead assumed the average portion of the UK population [33], which might differ from portions consumed by our obese participants.

On the other hand, self-perceived items like the TIS have been designed to detect differences between conditions in experimental studies [49]. However, some have claimed that reported changes are influenced by demand characteristics [50], with participants in more active conditions being more aware of study aims and so responding differently. Two previous studies counter the argument of demand characteristics by showing no difference between conditions for awareness of the study’s hypothesis or feelings of obligation to comply [17,21]. Further research is needed to improve our ability to measure change in dietary intake (eg, through more objective measures).

To our knowledge, our planning tool is the first fully automated system to test online if–then plans in the format of an interactive volitional help sheet. An advantage of our approach was that participants could choose more personally relevant situations [30] from the list, promoting a sense of autonomy [51]. However, a disadvantage is that the list did not include highly idiosyncratic situations that a participant might have entered through a free-text entry approach. Future studies could evaluate the relative impact of guiding participants to appropriate cues versus giving them complete autonomy.

As this was the first evaluation of a fully automated PT, we used a completers, per-protocol analysis, which, although limiting interpretation of the application of our results, allowed us to focus on the impact of the tool when used appropriately. Further research is needed to test the effectiveness of an implementation intention-based automated PT at a population level (via intention-to-treat analysis) over longer periods of time and to evaluate the impact of reminders [52-55].

Another advantage of the current study was that we assessed risk perceptions at two time points, giving us the opportunity to measure change in risk perceptions. Also, whereas previous research has used fictitious illnesses and hypothetical scenarios to communicate risk [23,41], our study risk corresponded to participants’ personal characteristics, making it more relevant. To the best of our knowledge this is the first study to combine implementation intentions with personally relevant health risk information through a Web-based medium.

### Conclusions

Web-based tools provide a good opportunity to present risk information and plan behavior change. In the present study, the HA risk message helped improve obese people’s awareness of risk relative to their age, and the PT reduced levels of perceived saturated fat intake. Future research is required to identify ways of matching motivational and volitional strategies to change behavior.

## Multimedia Appendix 1

Research protocol.

## Multimedia Appendix 2

Ethics committee approval document.

## Abbreviations

CG: control group
CVD: cardiovascular disease
FFQ: food frequency questionnaire
HA: Heart-Age
PT: planning tool
Q1: individual risk question
Q2: comparative risk question
TIS: 2-item scale
