# Supplementary material for: Web-Based Risk Communication and Planning in an Obese Population: Exploratory Study
Source: J Med Internet Res. 2011 Nov 24;13(4):e100. doi: 10.2196/jmir.1579 (PMC3278086; doi:10.2196/jmir.1579)
Supplement: Supplementary file 1 [file jmir_v13i4e100_app1.pdf]

Study Title: **Heart-Age and Planning in the Promotion of Saturated Fat Intake Reductions**

Study Number: **UCR2008-1016**

Date: 1st December 2008

Table of Contents

Part I: Summary

Protocol Summary

Study Schematic

Part II:

1. Study Overview

1.1 Background information
1.2 The Heart-Age Tool
1.3 Rationale for performing the study
1.4 Brief description of intervention
1.5 Summary of risks and benefits to the subjects

2. Study Objectives

2.1 Primary
2.2 Secondary

3. Study Design

3.1 Design and Rationale

4. Study Population

4.1 Total Number and Nature of Subjects
4.2 Subject Eligibility
4.3 Restrictions and Prohibitions

5. Study Treatments

5.1 Study Plan
5.2 Study Assessments
5.3 Study Duration
5.4 Dispensing and Accountability of Study Supplies

6. Premature Withdrawal

6.1 Withdrawal from the Study
6.2 Consequences of Missing Assessments, Withdrawals and Dismissals

7. Investigational Events

7.1 Endpoints
7.1.1 Primary
7.1.2 Secondary

8. Experimental evaluations

8.1 Biological Samples

9. Safety
9.1 Unilever Safety Clearance Requirements
9.2 Local Research Ethical Review Requirements
9.3 No Fault Compensation
9.4 Data Protection
9.5 COSHH
9.6 Head of Research
9.7 Microbiology

10. Serious Adverse Events

11. Statistical Considerations
11.1 Description of Statistical methods and interim analysis
11.2 Data Analysis and Plan of Efficacy
11.3 Sample size and power determination
11.4 Definition of Data and Analysis Population
11.5 Responsibilities for Statistical Analysis
11.6 Reporting of deviations of statistical plan

12. References

Part III: General Considerations for Conducting a Protocol

1. Ethical Consideration
1.1 Declaration of Helsinki
1.2 Good Clinical Practice

2. Investigators Responsibilities
2.1 Ethics
2.1.1 Declaration of Helsinki
2.1.2 Good Clinical Practice
2.1.3 Ethical Committee or Institutional Review Board
2.1.4 Informed Consent
2.1.5 Withdrawal of a Subject
2.1.6 Subject's Anonymity
2.2 Protocol Amendments
2.3 Case Report Form
2.4 Source Document Verification
2.5 Adverse Event
2.5.1 Adverse Event Definition
2.5.2 Unexpected Adverse Treatment Reaction
2.5.3 Causality
2.5.4 Adverse Event Severity Definition
2.5.5 Treatment & Follow-up of Adverse Events
2.6 Publication
2.7 Archiving
2.8 Audit

3. Sponsor Responsibilities
3.1 General Responsibilities
3.2 Monitoring and Contract Research Organisation Responsibilities
3.3 Quality Assurance & Quality Control
3.4 Archives
3.5 Final Report
3.6 Insurance

Part IV: Appendices

Appendix 1: **Study Schedule**
Appendix 2: **Example of the Online Informed Consent Form**
Appendix 3: **Example of the GP Letter**
Appendix 4: **Questionnaires**
Appendix 5: **Planning**
Appendix 6: **Recruitment Agency Screening**
Appendix 7: **HAPA Model Diagram**
Appendix 8: **Participant Debrief**

Part I: Protocol Summary

PROTOCOL SUMMARY

Title	**Heart-Age and Planning in the Promotion of Saturated Fat Intake Reductions**
Location	Online
Study Objective (s)	The aim of the study is to investigate the role of Heart-Age and Planning in the promotion of changes in saturated fat intake
Study Design	Randomised Control Trial
Study Population Sample Size	400 UK online users, women and men between the ages of 30-60.
Intervention	An Internet based study. Volunteers will be allocated into one of four different conditions: a) Heart-Age and Planning, b) Heart-Age, c) Planning, d) Control Group. At Time 1, all subjects will be asked to fill out a set of questionnaires. At Time 2, 1 week later depending on the condition that subjects have been assigned to, they will be asked to go through Heart-Age and Planning, Heart-Age or Planning alone or to fill out a number of questionnaires on social-cognitive determinants of saturated fat intake (control group). Two weeks later, subjects will come back to the website and report on changes in their saturated fat intake and a number of social-cognitive determinants.
Study Endpoints	The primary endpoint is:<br>a) Intervention impact on self-reported saturated fat intake<br><br>The secondary endpoint is:<br>a) Intervention impact on self-efficacy, risk perceptions, intentions, outcome expectancies as measured in the HAPA model
Duration of Study	Time 1: 30mins<br>Time 2: up to 30mins (depending on allocated condition)<br>Time 3: 30mins

Part I: Study Schematic:

Phase 1	Phase 2	Phase 3
1) Subjects' Online Screening<br><br>2) Signing Online Consent Form	1) Random allocation of subjects in one of four conditions:<br>a) Heart-Age and Planning<br>b) Heart-Age<br>c) Planning<br>d) Control Group	
3) Filling out Questionnaires on:<br>a) Dietary Knowledge,<br>b) Saturated Fat Intake and<br>c) Psychological Determinants	2) Filling out Questionnaires on:<br>a) Saturated Fat Intake<br>b) Social Cognitive Determinants<br>c) Feedback on the Information Received	1) Filling out Questonnaires on:<br>a) Saturated Fat Intake<br>b) Social Cognitive Determinants<br>c) Feedback on Information Received
4) Re-direction to Virtual Surveys' website	3) Re-direction to Virtual Surveys' website	2) Re-direction to Virtual Surveys' website<br><br>3) Reimbursement of Subjects and entrance into Prize Draw

1. Study Overview

1.1 Background Information

A healthy diet, low in saturated fat and high in fiber, is a popular medical recommendation in preventing cardiovascular disease (CVD). These type of recommendations would be particularly effective for high CVD risk groups i.e. smokers and obese (Renner et al, 2000).

The likelihood of someone changing their behaviour to reduce their saturated fat intake and future risk of developing heart-problems can be enhanced by:

- Interventions designed to change ***risk perceptions.***
- Forming specific ***plans*** on how to change behaviour.

Effective communication of heart-health messages is of foremost importance. What is becoming quite clear is that the general population is unaware of true risk of cardiovascular disease and find difficult to understand CVD risk messages. For example, almost one half of adults in USA have literacy levels that impede their ability to understand health information and apply it to behaviour change (Goldman et al, 2006).

Flora/Becel has launched a CVD risk calculator, the Heart-Age, which helps consumers understand their risk to cardio metabolic related diseases. This is an interactive tool designed to calculate how old a person's heart is compared to their chronological age (relative risk). The tool, which has a strong scientific base, was developed using Framingham's mathematical method called the Framingham Risk Score.

The Heart-Age system was previously compared online against a standard CVD risk score (risk seen as a percentage). Subjects who interacted with the Heart-Age system were more emotionally responsive to it than the standard CVD risk score. Subjects at higher actual CVD risk going through the Heart-Age were also more likely to perceive their risk accurately. This was especially true for the younger subjects (30-45 years).The relationship between perceived risk and motivation to make lifestyle changes was further mediated by emotional responsiveness.

This study was significant in shedding light on the Heart-Age tool's capability to motivate subjects via the instigation of emotional reactions. There are still lots of questions left unanswered. Though personalisation of risk communication through the Heart-Age score seemed to help people better understand risk, its effects were not assessed in a person's actual behaviour. Does the Heart-Age tool play a significant role at both the motivational and the volitional process?

1.2 The Heart-Age Tool

The Framingham Heart Study (FHS), which started in 1948, is a prospective epidemiological study, undertaken in Framingham, Massachussets into the causes of

heart disease. The age of the original cohort ranged from 28-62 years old at the outset of the study and all subjects were free of CHD. Every two years they were followed up for measurement of biological factors and to determine the incidence of heart disease, cancer and other disorders. In 1971, the offspring of this original cohort were enrolled (The Framingham Offspring Study, FOS) enabling the study to track the influence of family history and ultimately genetic influences. The Framingham Heart Study was instrumental in establishing the relationship between specific risk factors, such as cholesterol and hypertension, and risk of heart disease. Furthermore, as the data-set grew in size, the confidence in the findings enabled the accurate assessment and quantification of CHD risk which we see being used today.

Recently, there have been a number of coronary risk calculators providing the public with access to tools that define personal risk of heart disease (http://www.nhlbi.nih.gov/about/framingham/riskabs.htm). However, many health professionals find it difficult to assimilate multiple risk factors into accurate assessment and explanation of CVD risk to their patients (Ben Van Steenkiste et al, 2007).

Unilever collaborated with the Boston University Statistics and Consulting Unit in an effort to: a) identify the factors that increase people's risk of heart disease in the world-famous Framingham Heart Study, and b) find other appropriate ways to improve the presentation of risk to the wider population. This involved a number of activities from statistical re-analysis of the data to consumer studies. The aim was to identify the most motivating format for a (Framingham) heart disease risk assessment that may be used directly with consumers and in a way that motivates behaviour change.

This led to the development of the 'Heart-Age' tool, an interactive tool designed to calculate how old a person's heart is compared to their chronological age. The tool, which has a strong scientific base, was developed using Framingham's mathematical method called the Framingham Risk Score. People input their levels of blood pressure, blood cholesterol, whether or not they are a smoker or a diabetic – all factors that increase their risk of heart disease. The tool has been **endorsed by the World Heart Federation** and also by heart foundations and GP associations in each country in which it has been launched.

1.3 Rationale for Performing Study

The present study is a variant of a previous study conducted by Corporate Research and approved by the Ethics Committee at the beginning of this year. Heart-Age, which was found to be more beneficial than the standard CVD risk calculator on helping people accurately perceive their risk of future heart-disease will be now coupled with planning to see whether this could lead to changes in actual behaviour. The behaviour chosen will be saturated fat intake and the target population will be overweight/obese subjects. Will the Heart-Age in conjunction with Planning play a significant role at both the motivational and the volitional process? To achieve this, it is of foremost importance to base a study on an appropriate psychological model that fits the research needs.

Health Behaviour Change: The Health Action Process Approach

The emphasis of most current psychological models has been placed in identifying a number of factors that impact intentions (motivational phase), leaving the translation between intentions into action less advanced (volitional phase). In the present study, an effort was made to choose a model that not only focuses on motivation but also on the translation of motivation into behaviour. The Health-Action Process Approach (HAPA, Schwarzer 1999, 2001) pays particular attention not only on determinants of intention but also on what happens once someone formulates the intention to change their behaviour. For a diagram of the HAPA model please see Appendix 8.

Three dimensions have been identified in the HAPA model as being influential in affecting people's motivation to change. These are namely *risk perceptions*, *outcome expectancies* and *self-efficacy*.

Before people change their habits they need to become motivated to do so. Perceiving a *health threat* seems to be an important prerequisite to adopting health behaviours. Scare tactics have not been found to be too effective. In general, initial perceptions of risk seem to put people on the right track to forming an intention to change but later other factors play a more significant role.

Outcome expectancies can be quite influential in the motivational phase when the person is considering the pros and cons of carrying out the behaviour. Similarly, perceived *self-efficacy,* the belief in one's ability to perform a behaviour, is critical when approaching a difficult behaviour. Two different types of self-efficacy will be tested in the current model: a) action self-efficacy and b) maintenance self-efficacy. Action self-efficacy is instrumental in the pre-actional phase, since people high on this dimension seem to be confident in imagining success and are more likely to initiate a new behaviour. Maintenance self-efficacy, on the other hand, deals with the confidence displayed by someone in dealing with barriers that arise once the behaviour has been initiated. The role of these constructs in the motivation stage has been tested before in the context of some preventive behaviours such as exercise and nutrition (Fuchs, 1997; Schwarzer & Fuchs, 1996; Luczcynska & Schwarzer, 2003).

Translating Intentions into Action

If we are to be talking about behaviour change we need to be able to explain behaviour but also change it. The Heart-Age could be more influential than a standard CVD risk score into formulating appropriate risk perceptions and motivating change. The inclusion of action plans also known as implementation intentions can bridge the gap between intention and behaviour.
Research on action plans is not new. For example, Leventhal et al (1965) suggested that fear appeals can facilitate change only when they are combined with specific instructions on where, when and how to perform them. Ajzen et al (2005) also indicated that planning is effective because it creates a sense of commitment to the intended behaviour. A meta-analysis of 94 studies (Gollwitzer & Sheeran, 2006) showed that implementation intentions had a positive effect of medium to large magnitude on goal achievement. It also provided some evidence that implementation intentions can change dietary behaviours.

Fine tuning of planning is essential to goal attainment. Though previous research was more concerned with proving the value of using action plans, more research is now

needed on the mechanisms that make an action plan effective. A number of different formats have been used in the past. In experiments, subjects have been asked to form a specific plan in as much detail as possible paying particular attention to the situation in which they will implement these plans. For example, '…Please write below when, where and how you will make an appointment…' (Sheeran & Orbell, 2000). Though important these statements fail to create a strong cue-response relationship.

'If…then' statements could be more beneficial than the more global goal intention instructions in two ways. Firstly, 'if' statements make the critical situation more accessible to the user and thus shifting detection to the situation once encountered in the environment. Secondly, the 'then' component of the plan creates a stronger link between the situational cue and the goal-directed response, automatically initiating action when the critical situation is encountered. For example, Oettingen et al (2000) compared the effects of forming a goal intention alone to a goal intention furnished with an 'if..then' format. Whereas in both cases a concrete situation was specified, subjects in the 'if..then' condition adhered more closely to the time specified in their plan. Chapman et al (2008) also found that 'if..then' statements increased significantly fruit and vegetable consumption compared with the more global goal intention format in a student population.

1.4 Brief Description of Intervention

The primary aim of the current study is to assess Heart-Age's motivational properties and its combined effects when used with planning on subjects' saturated fat intake.

Four sets of subjects will enter the online study site at three different times. In specific at **Time 1**, subjects will be invited to participate in the study by an online recruitment agency, which will also carry out the screening process. Once, subjects have been accepted in the study they will be asked to fill out three questionnaires: a) a self-reported dietary intake questionnaire, b) a self-reported questionnaire on various social-cognitive variables. At **Time 2**, which will be a week later, subjects will be randomly allocated into one of four conditions. In the most advanced condition subjects will go through the Heart-Age tool and then they will be asked to form a concrete plan on how to reduce saturated fat intake over the next two weeks. In the second and third conditions, subjects will be presented either with the Heart-Age tool or a planning session. There will be a control group, who will fill out questionnaires at pre-study, main study phase and post-study. At **Time 3**, subjects will be invited to fill-out again the same dietary measures of food intake and the questionnaire on the social-cognitive determinants. Study subjects will be rewarded for their participation in the study at Time 1 and 3 and will enter a prize draw of £200 in Amazon vouchers. This will be upon completion of study phase 3.

1.5 Summary of risks and benefits to the subjects

Benefits

There are a number of benefits to the subjects:

a) Heart-Age is viewed as a tool to help people take care of their hearts before it is too late. It may help identify those subjects with high risk factors (e.g. smokers, obese) that are predisposed to a higher lifetime risk of CVD despite being at low short term risk of developing CVD.

b) Heart-Age could be also viewed as a decision making tool that might help reduce consumers' complacency, increase awareness and helps them embark on future health decision making.
c) Starting a two-way interaction with consumers is crucial if we want consumers to start taking personal responsibility for their health. This study will be one of the few to attempt to start a two-way dialogue with consumers on risk communication and dietary change.

Risks

The only potential risk is that some subjects may get unnecessarily worried by their Heart-Age. This is due to the fact that subjects might be presented with higher than expected Heart-Age score.

To minimise this risk a number of actions have been taken:

a) The Heart-Age tool as a whole is a valid predictor of CVD risk and the model is soon to be published in a high impact cardiology journal. It has also been endorsed by the WHF and the Heart Foundations in each country in which it has been launched.

b) Feedback messages on the site have been designed in such way as to minimise potential excess worry of subjects. This heart-healthy advice has been seen by the World Heart Federation (WHF).

c) In all cases, subjects are provided with a message motivating them to visit their GP IF they want to embark on a lifestyle change. Please see Table 1 for a presentation of the different type of messages depending on subjects' absolute risk score. Diabetics will be the only high risk group that will not receive a letter to take to their GP since their health professional would be already aware of their health condition.
d) The maximum 'Heart Age' that an individual can receive will be capped at 15 years older than their actual age.
e) Subjects with one elevated risk factor (i.e. being obese; BMI>30) or high absolute CVD level (>30%) can print and take a letter to their GP. This is no different in reality to the current policy in face-to-face clinical studies, where subjects with e.g. high blood pressure are presented by a study nurse with a letter to take to their GP with no further advice.
f) The Heart Age tool does not suggest that this is a medical advice tool and clearly states that any medical issue should be discussed with a Health Care Practitioner.
g) The external recruitment agency will be provided with a list of the most frequently asked questions and answers that have been used on the existing Flora-Becel website to be passed on to subjects (if requested). If there are any questions that Virtual Surveys find difficult to handle they will get in touch with the study coordinator.

Table 1. Type of Participant Messages and Absolute Risk Score

Absolute Risk Score	Type of Message	BMI	GP letter
<15%	One of your risk factors appears to be elevated. We would advise you to talk to your doctor about it. Before embarking on any lifestyle changes it is advisable to speak with your doctor.	>30	Yes
15-30%	From your answers, one of your risk factors appears to be elevated. Next time you are at the doctors perhaps make time to discuss your heart health. In addition, before embarking on any lifestyle changes it is always advisable to speak with your doctor.	>30	Yes
>30%	From your answers we would advise you to talk to your doctor about your heart health and possible ways to improve upon it	≥25	Yes

In the previous study conducted at the beginning of 2008, the pros seemed to have outweighed the cons. Subjects did not seem to get unnecessarily anxious by the information received and no-one asked Virtual Surveys for additional clarifications on the Heart-Age.
There were some really positive comments received

e.g.
'….No this was a good survey. I have already started to make changes since the New Year and this has encouraged me to carry on…'

'..I have recently undergone an E.C.G. and everything was fine, however, I do understand the risks involved in smoking. It was very interesting and informative and I intend to take an immediate action to remedy the results. Thank you…'.

'…Could do with something like this coming in on a regular basis to help keep me motivated!...'

There were also tips and more neutral comments offered such as:

'…Very brief for the type of questionnaire and didn't really take into account other factors, i.e. vitamin usage and more personal and accurate information on diet.exercise. A very 'overview' test…'

'…I feel that the psychological factors involved are under represented…'

2. Study Objectives

2.1 Primary Objectives

Objectives:
The primary aim of the present study is to test the effects of the Heart-Age and Planning on Motivation to Change and Saturated Fat Intake over a period of two weeks. It is hypothesised that Heart-Age will exert its effect on motivation and planning on behaviour and that in combination they would fare better than if used independently.

To assess the above, two questionnaires will be issued:

1. Margetts et al (1989) self-report index of food
2. A two item self-perception of saturated fat intake to account for any reporting and demand characteristic biases

2.2 Secondary Objectives

The secondary objectives of this study is to evaluate the intervention effects on a number of social-cognitive variables e.g. intentions, self-efficacy, planning, outcome expectancies. The constructs of the HAPA model namely risk perceptions, outcome expectancies, self-efficacy and planning will be measured via questionnaires. For a diagram of the HAPA model see Appendix 8.

3. Study Design

3.1. Design and Rationale

This is a randomised, between-groups study designed to assess the difference in saturated fat intake between four different experimental conditions; a) Heart-Age and Planning, b) Heart-Age, c) Planning and d) Control Group. Subjects will be randomly allocated to one of these conditions and their responses will be measured by self-reported questionnaires. Table 2 presents the breakdown of subjects in the different conditions. Allocation to the tools will be stratified to balance by age group (30-45 years or 46-60 years), and gender.

Table 2. Breakdown of target participant numbers in the four conditions

HA+Planning		Heart-Age		Planning		Control Group	
Age	Gender	Age	Gender	Age	Gender	Age	Gender
30-45 years (N=50)	*Males (25)*	*30-45 years (50)*	*Males (25)*	*30-45 years (50)*	*Males (25)*	*30-45 years (50)*	*Males (25)*
	Females (25)		*Females (25)*		*Females (25)*		*Females (25)*
46-60 years (N=50)	*Males (25)*	*46-60 years (50)*	*Males (25)*	*46-60 years (50)*	*Males (25)*	*46-60 years (50)*	*Males (25)*
	Females (25)		*Females (25)*		*Females (25)*		*Females (25)*

4. Study Population

4.1 Total Number and Nature of Subjects

We will over-recruit at Time 1 to be able to take into account any drop-outs likely to occur over the study period. The aim will be to end up with 400 (or more) participants at Time 3. 100 subjects will be used for each cell of a 2x2 factorial design with the factors 'use of the Heart-Age tool' and 'use of planning'. Thus 200 subjects will use the Heart-Age site, 100 of these will plan how to reduce their saturated fat intake and the other 100 will not. Of the remaining 200 subjects, 100 will also be asked to plan how to reduce their saturated fat intake. The final 100 will act as a Control group (neither using the Heart-Age tool nor planning how to reduce their saturated fat intake).

Subjects will be recruited from both genders, be 30-60 years old and will have a high level of at least one risk factor for CHD (normally obesity). This inclusion of subjects with at least one risk factor has been deliberate. It is believed that these subjects,

though at no immediate risk of developing heart disease, will be more likely to benefit from presentation of information on their heart-health. If the information is motivating enough then they will hopefully decide to take some action to maintain or improve their future heart-health. Obese and overweight people are also expected to take into consideration their weight when making health-related judgements. Thus they should consider themselves as being at higher risk than people of average weight (Renner et al, 2000).

4.2 Subject Eligibility
Subjects will be screened by use of an online questionnaire assessing weight/height, age, gender, pre-existing cardiovascular disease and cancer.

Exclusion Criteria:
- BMI <29 (Body Mass Index)
- <30 years old
- >60 years old
- Pregnant women
- Diagnosed with cancer
- Diagnosed with a heart-condition (heart-attack or angina) – this is because algorithms predict risk of first CVD event, not recurrent events.
- Any other chronic disease of the major organs (e.g. kidney failure)
- Not willing to sign online consent form
- Not literate in use of computer and the internet
- Not being able to print at home or at work

Inclusion Criteria
- BMI ≥29
- 30-60 years old
- Male or female
- Not diagnosed with a heart-condition (heart-attack or angina)
- Not diagnosed with cancer
- Willing to sign the Informed Consent Form
- Computer and internet literate
- Being able to print at home or at work

4.3 Restrictions and Prohibitions

There will be no restrictions or prohibitions placed once subjects who have entered main study-phase.

5. Study Treatments

5.1 Study Plan

The flow of the different study phases is seen in Figure 1 below.

Figure 1 – Study Flow

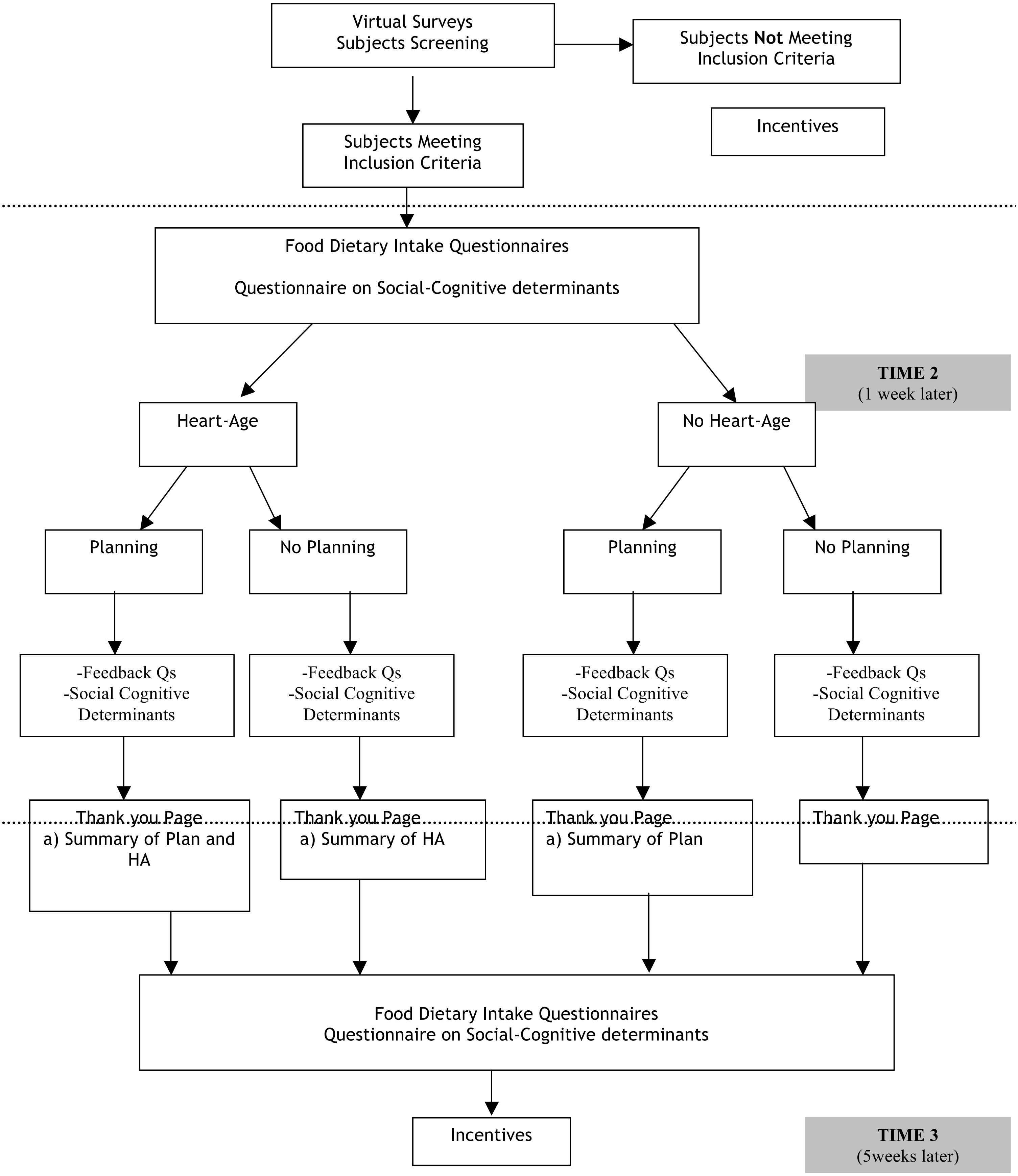

Phase 1 – Recruitment and Screening
Recruitment will be conducted by Virtual Surveys (http://www.virtualsurveys.com/s), an online recruitment agency, to cost effectively reach a specific target sample within the UK population. Virtual Surveys have their own online panel and a structured script will be used to screen individuals and exclude any people who do not meet the inclusion criteria (Appendix 6). Once subjects meet the inclusion criteria, they will be acquainted with the study procedures and asked to read an online informed consent form. They will be requested to click on a number of check boxes to make sure they have read and understood the information before they proceed to the online questionnaires. Participants will out items on their perceived saturated fat intake and social-cognitive determinants.

Phase 2 – Main study
In the main study phase, subjects will be randomly allocated into one of four different conditions: a) Heart-Age and Planning, b) Heart-age, c) Planning and d) Control Group. Those subjects going through Heart-Age will fill out a CVD assessment questionnaire. A paper copy version of the online questionnaire is found in Appendix 4. Once the CVD risk assessment questionnaire has been filled out then subjects will receive a Heart-Age score followed by brief advice on a number of lifestyle related aspects (Figure 2). Subjects in the planning condition will identify a list of situations where they would like to change their saturated fat intake. They will then be asked to match these situations with a list of behaviours. They would be requested to choose pairs of situations and behaviours that they want to change rather than ones that they are already doing (Appendix 5).
All subjects will be invited to answer a number of questions on their perceptions of the information received, intentions to make lifestyle changes, feelings of self-efficacy and expected outcomes. Subjects in the control group will only receive the questionnaires.

Phase 3 – Study End
In the last phase, subjects will come back to the online study site to fill out a number of questionnaires. Upon completion of study, subjects will be debriefed (Appendix 8) and directed back to the Virtual Surveys site to receive payment (£15) for participation in the study and also be put in the prize draw of Amazon vouchers (£200).

Figure 2. Heart-Age Risk Score

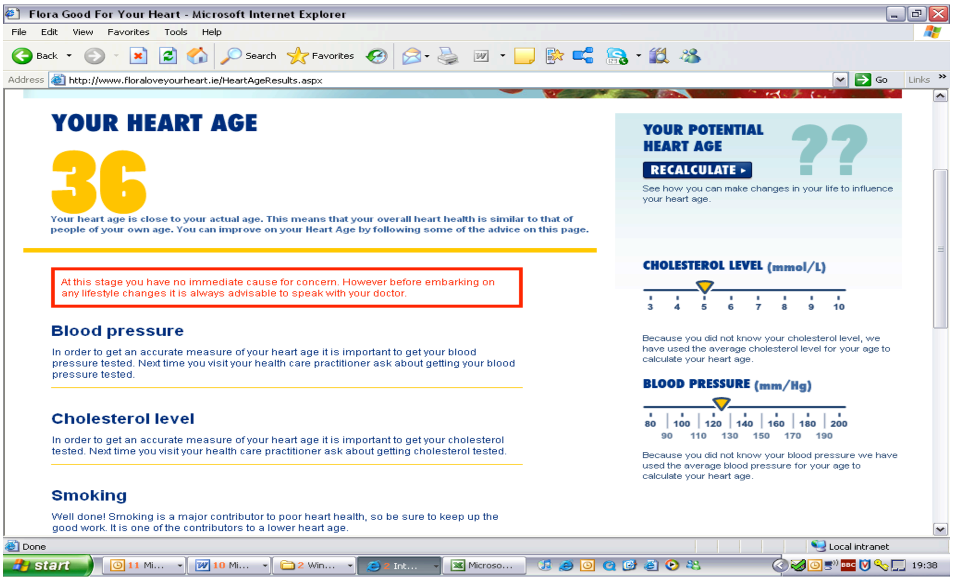

5.2 Study Assessments

Subjects will complete a number of questionnaires.

<u>Primary Outcome</u>

<u>Saturated Fat Intake</u>
Self-reported amount of saturated fat intake will be measured by Margetts et al (1989) 63 item questionnaire and a two-item self-reported measure. The Margetts, Cade & Osmond (1989) questionnaire is a validated self-report index of food. The frequency of consumption of 63 common foods over the previous month using a 6-point-scale is assessed (two or more times per day; three to five times per week; one to three times per month; rarely or never). The scale has good test-retest reliability (r=.61; Armitage & Conner, 1999).
Saturated fat intake will be also measured by a two item self perception questionnaire.

<u>Secondary Outcomes</u>

<u>Self-Efficacy/ Expected Outcomes/ Planning</u>
There will be a second questionnaire that will cover a range of psychological determinants. The pre-actional and maintenance self-efficacy, expected outcomes, and planning measures were adapted from Renner & Schwarzer's (2007) and Schwarzer's (2008) publication on assessment of the Health Action Process Approach Model (HAPA) and from Sienhotta et al.'s (2005) paper on physical activity.

<u>Intentions</u>
A measure of intention to saturated fat intake was adapted from Prestwich et al.'s study (in press) ('Over the next two weeks I intend to reduce my saturated fat intake'). A more detailed list of intentions was designed and tested with subjects for their external validity. The purpose has been to measure a number of saturated fat intake behaviours and add them up to get a total score of saturated fat intake. At follow-up, there were two further questions on participants' intentions to assess their cholesterol and blood pressure over the next month.

Reactions to the Intervention
Subjects' opinions of the intervention were adapted from Bourdeaudhuij & Brug (2000), and Brug et al (1998). Two extra items were included on emotional reactions to the information presented ('I felt that the information…..was worrying', …'a wake-up call').

Table 2 below shows the questionnaires to be filled out at the three different study times.

Table 2. Study Assessments at the three different study times

TIME 1	TIME 2	TIME 3
Intentions (saturated fat)	Intentions (saturated fat)	x
x	x	Intentions (BP/cholesterol)
Outcome Expectancies	Outcome Expectancies	x
Action SE	Action SE	x
x	Maintenance SE	Maintenance SE
Risk Perceptions	Risk Perceptions	x
FFQ	x	FFQ
x	x	Planning
x	Beliefs on Info received (wake-up call, credibility etc)	Beliefs on Info received (wake-up call, credibility etc)

5.3 Study Duration

This study requires three online visits to a web site. The same subjects will not be contacted in the future to fill out any other information on these risk tools. The whole process, including screening and main study phase, should not last more than 3hours.

5.4 Dispensing and Accountability of Study Supplies

N/A

5.4 Continuation of Treatment

The subjects will not have access to the Heart-Age after completion of the study. The current link will be only designed to be used during the study.

6. Premature Withdrawal

6.1 Withdrawal from the Study

If a subject meets the inclusion criteria but decides to withdraw mid-way through the study then no data will be uploaded in the system and a request will be made to virtual surveys for this person to be replaced by another subject with the same characteristics.

6.2 Consequences of Missing Assessments

There are three assessment points in this study. Virtual Survey has suggested a 2/3 drop out from one study phase into the next. To account for this, 900 subjects will be recruited to end up with 400 at the end of the study.

7. Investigational Events

7.1 Endpoints

7.1.1 Primary
The primary endpoint is:
a) Self-Reported Saturated Fat Intake

7.1.2 Secondary
The secondary endpoints are:
a) Intervention effects on self-efficacy, risk perceptions, intention, outcome expectancies as measured by the Health Action Process Approach Model.

8. Experimental evaluations

8.1 Biological Samples
No biological samples are being collected in this study and there are no invasive measures.

9. Safety
Subjects will be recruited only when the appropriate approvals have been granted.

9.1. Unilever Safety Clearance Requirements
N/A

9.2. Local Research Ethical Review Requirement
The authorised protocol and other appropriate documentation will be subject to the approval of Independent Ethics Committee in Colworth. The study will not proceed without the written approval of the Ethics Committee and any recommendations for change or additions will be discussed and necessary changes made. Finally the Research Ethics Committee will be sent a summary of the study findings for their information.

9.3. No Fault compensation
In the unlikely event of any subject suffering any significant deterioration in health or wellbeing arising as a result of taking part in this study, Unilever has undertaken to compensate them without having to prove that anyone involved in this study has been negligent. Any dispute will be referred to an arbitrator.

9.4. Data Protection
Information relating to this study will be kept confidential and the subjects' right to check personal data will be fully protected in accordance with the Data Protection Act 1998. Use of Case Record Forms is not applicable in this study since all information will be gathered via an online system. Each subject will be allocated with a unique ID at the recruitment stage (by Virtual Surveys) which will remain the same until the end of the study. Subjects will be responsible for entering their data on the system and no personal details such as (first name, last name and email addresses will be asked of the subjects). However, subjects will be required to fill out personal health information, gender, date of birth into the website in order to receive feedback at the end of the study.

The subject may choose not to provide certain information, but this may mean they will not be able to complete the study and their data will be replaced by new

participant data. The site will not contact the user and Unilever Corporate Research will not knowingly sell, rent, or trade the personal information collected.

All web-based interaction data will be stored for a period of five years in a secure central database hosted by Tessella Services on behalf of Unilever. Data files on the mainframe will be backed-up automatically. All data files will be backed-up and stored in a secure location.

9.5 COSHH
N/A

9.6 Head of Research
Once all of the study approvals have been collected, the Corporate Clearance form will be signed off by the Head of Corporate Research.

9.7 Microbiology
N/A

10. Serious Adverse Events

Any adverse event that is considered SERIOUS must be reported within one working day by the investigator to the sponsor/study co-ordinator (for definition see Protocol part III; section 2.5 "Adverse Event").

11. Statistical Considerations

11.1 Description of Statistical methods and interim analysis

This is a randomised, stratified, between-groups study using a 2x2 factorial design with the factors 'use of the Heart-Age tool' and 'use of planning'. The primary endpoint is the change in saturated fat intake in participants allocated to the four different experimental conditions (Heart-Age and Planning, Heart-Age, Planning and Control Group). Participants will be randomly allocated to one of these four conditions, stratifying for age group (30-45 years or 46-60 years) and gender. Their saturated fat intake will be measured at baseline and the completion of the study (4 weeks after enrolment). The statistical analysis will compare the efficacy of the four treatment groups via the change in saturated fat intake between the baseline and final diet assessments. There will be no interim analyses.

11.2 Data Analysis and Plan of Efficacy

A full statistical analysis plan will be formulated in collaboration with Peter Murray from the Unilever Statistics Group, Colworth Park. The effect of the design factors will be determined by comparing the proportion of subjects who have reduced their saturated fat intake, using a logistic regression model which includes the two design factors and their interaction. Further analysis on the actual mean level of reduction may also be carried out, using an analysis of variance model including the design factors and their interaction (data transformations may be required to ensure normality assumptions are not violated). The stratification variables will be included in the models as will other covariates found significant at the 0.05 level. Comparisons between levels of a design factor will all be reported using two-sided significance

levels (i.e. making no prior assumption about which treatment group will be most effective).
If there are any relevant differences at baseline measurements between the four groups, we will adjust the outcome of these factors (e.g. diet knowledge, history of saturated fat intake, socio-economic status). Separate analysis of the effects of modifiers and mediators will be conducted in order to gain a better insight as to who benefits from the intervention.

11.3 Sample size and power determination

The primary endpoint of saturated fat intake reduction will be assessed by calculating the proportion of subjects who reduced that intake within each of the four treatment groups and comparing these proportions. Comparisons between groups will then be based on 100 subjects per group. Comparisons between the factors (i.e. 'use of the Heart-Age tool' and 'use of planning') will be based on 200 subjects.

A sample size of 200 subjects per cell will allow a difference in proportions of correct responses of approximately 0.08 to be detected at a two-sided 0.05 significance level with power of 0.8, assuming that the lower proportion is around 10% of subjects (i.e. the 10% of non-Heart-Age users their saturated fat intake, then we would need 18% of Heart-Age users to reduce to have a good chance of detecting a significant difference).

A sample size of 100 subjects per cell will allow a difference in proportions of correct responses of approximately 0.12 to be detected at a two-sided 0.05 significance level with power of 0.8, assuming that the lower proportion is around 10% of subjects (i.e. the 10% of Heart-Age non-planners reduce their saturated fat intake, then we would need 22% of Heart-Age planners to reduce to have a good chance of detecting a significant difference).

11.4 Definition of Data and Analysis Population

Data will be recorded by subjects, captured via the on-line system and will be held in compliance with the UK Data Protection Act. Each subject will be identified by a unique ID number assigned to them
The original data will be stored in the computer (but no hard-copies will be kept since this is an online study) and will be filed at Unilever for a minimum of five years from completion of the study.

11.5 Responsibilities for Statistical Analysis

All statistical analyses will be performed by the Unilever Statistics Group, Colworth Park

11.6 Reporting of deviations of statistical plan

Any deviations of the statistical plan will be reported in the analysis.

12. REFERENCES

Armitage, C.J., & Conner, M (1999). The theory of planned behaviour: Assessment of predictive validity and perceived control. *British Journal of Social Psychology,* 38, 35-54.

Bourdeaudhuij, I.D., & Brug, J. (2000). Tailoring dietary feedback to reduce fat intake: an intervention at the family level. *Health Education Research: Theory and Practice,* 15 (4), 449-462.

Brug, J., Glanz, K., Assena, P., Kok, G., Breukelen, G.J.P., (1998). The impact of computer-tailored feedback and iterative feedback on fat, fruit and vegetable intake, *Health Education and Behaviour,* 25 (4), 517-531.

Chapman, J., Armitage, C.J., & Norman P. (2008). Comparing implementation intention interventions in relation to young adults' intake of fruit and vegetable. *Psychology and Health,* 1-16

Gollwitzer, P. M & Sheeran P (2006). Implementation intentions and goal achievement: a meta-analysis of effects and processes. *Advances in Experimental Social Psychology,* 38, 69-111.

Jackson C., Lawton, R., Knapp., P., Raynor, D., Conner, M., Lowe, C et al (2005). Beyond intentions: Do specific plans increase health behaviours in patient primary care? A study of fruit and vegetable consumption, *Social Science and Medicine,* 60, 2383-2391.

Leventhal, H., Singer R ., & Jones S. (1965). Effects of fear and specificity of recommendation upon attitudes and behaviour. *Journal of Personality and Social Psychology,* 2, 20-29.

Luszczynska, A& Schwarzer, R (2003). Planning an self-efficacy in the adoption and maintenance of breast self-examination: a longitudinal study on self-regulation cognitions, *Psychology and Health,* 18 (1), 93-108.

Margetts, B.M., Cade, J.E., & Osmond, C. (1989). Comparison of a food frequency questionnaire with a diet record, *International Journal of Epidemiology,* 18, 868-873.

Oettingen , G., Honig, G., Gollwitzer, P. M. (2000). Effective self-regulation of goal attainment. *International Journal of Educational Research*, 33, 705-732.

Orbell, S & Sheeran, P (2000). Motivational and volitional processes in action initiation: a field study of the role of implementation intentions. *Journal of Applied Social Psychology,* 30, 780-797.

Prestwich, A. (in press). Crossing two types of implementation intentions with a protection motivation intervention for the reduction of saturated fat intake: A randomised trial.

Prochaska J.O., Velicer, W.F., DiClemente, C.C., & Fava, J.L. (1988). Measuring the processes of change: Applications to the cessation of smoking. *Journal of Consulting and Clinical Psychology,* 56, 520-528.

Renner, B; Knoll N., & Schwarzer R. (2000). Age and body make a difference in optimistic health beliefs and nutrition behaviours, *International Journal of Behavioural Medicine*, 7(2), 143-159.

Rossi, S.R., Greene, W.G., Rossi., J.S., Plummer., B.A., Benisovich., S.V., Keller, S., Velicer, W.F., Redding, C.A., Prochaska, J.O., Pallonen, U.E., & Meier, K.S. (2001). Validation of decisional balance and situational temptations measures for dietary fat reduction in a large school-based population of adolscents. *Eating Behaviours,* 2, 1-18

Schwarzer, R (1999). Self-regulatory processes in the adoption and maintenance of health behaviours. The role of optimism, goals and threats. *Journal of Health Psychology*, 4, 115-127.

Schwarzer, R (2001). Social-cognitive factorsin changing health-related behaviours. *Current Directions in Psychological Science*, 10, 47-51.

Schwarzer, R., Sniehotta, F. F., Lippkie, S., Luszynska, A., Scholz U., Schuz B., Wegner M., Ziegelman, J (2003). On the assessment and analysis of variables in the health action process approach: conducting an investigation. In http://web.fu-berlin.de/gesund/hapa_web.pdf

Renner, B., & Schwarzer, R.(2007). Risk and Health Behaviours In www.gesundheitsrisiko.de

Schwarzer, R .(2008). Modelling health behaviour change: how to predict and modify the adoption and maintenance of health behaviours, *Applied Psychology: An International Review,* 57 (1), 1-29.

Sniehotta, F. F., Scholz, U., & Schwarzer, R. (2005). Bridging the intention-behaviour gap: Planning, self-efficacy, and action control in the adoption and maintenance of physical exercise, *Psychology and Health*, 2, 143-160.

Part III: General Considerations for Conducting a Protocol

1. Ethical Considerations

1.1 Declaration of Helsinki

The current revision of the Declaration of Helsinki is the accepted basis for clinical study ethics, and must be fully followed and respected by all engaged in research on human beings. Any exceptions must be justified and stated in the protocol. Independent assurance that subjects are protected can only be provided by an ethics committee/institutional review board and freely obtained informed consent.

1.2 Good Clinical Practice

Good clinical practice is a standard for clinical studies, which encompasses the design, conduct, monitoring, termination, audit, analyses, reporting and documentation of the studies. It ensures the studies are ethically justified and scientifically sound, and that the clinical properties of the

diagnostic/therapeutic/prophylactic product under investigation are properly documented. Refer to section 2.1.2 for further guidance.

2. Investigators Responsibilities

It is the responsibility of the investigator(s) to conduct the study according to the protocol and to ensure that (s) he has the subject availability to conduct the study within the period defined in the study protocol.

2.1 Ethics

2.1.1 Declaration of Helsinki

It is the responsibility of the investigator(s) to ensure that the study is conducted in full conformance with the principles of the current Revised Version (1989) of the Declaration of Helsinki.

2.1.2 Good Clinical Practice

It is the responsibility of the investigator(s) to ensure that the study is performed in accordance with Unilever's standards for the conduct of Human Trials and according to all local laws and regulations concerning Human studies. It is observed that due to the nature of Unilever human trials these do not qualify as Clinical Trials and as such, are not obliged by law to follow EU Directive 2001/20/EC (Clinical Trials Directive) or EU Directive 2005/28/EC (GCP Directive). However, Unilever Human Trials where possible operate to the spirit of these Directives in order to ensure best practice.

2.1.3 Ethics Committee or Institutional Review Board

It is the responsibility of the investigator(s) to submit a copy of the protocol and consent form to an ethics committee/institutional review board in order to obtain independent approval to conduct the study. Ethics committee/institutional review board approval must be obtained before the study is started. The approval of the ethics committee/institutional review board must be sent in writing, to the investigator(s). The Ethics Committee approval letter must mention the Ethics Committee members and their function.

2.1.4 Informed Consent

It is normally the responsibility of the investigator(s) to obtain informed consent from each subject participating in the study, after explanation of the aims, methods, benefits and potential hazards of the study. Since this study is solely conducted online the aims, benefits and potential harms to the subjects will be explained online (Appendix 2). The consent must and will be obtained before any study-specific procedures are performed.
It must be made completely and unambiguously clear to each subject that they are free to refuse to participate in the study, or that they can withdraw their consent at any time and for any reason, without incurring any penalty or withholding of treatment on the part of the investigator.

2.1.5 Withdrawal of a Subject

N/A

2.1.6 Subject's Anonymity

The investigator(s) must ensure that the subject's anonymity will be maintained. On all documents, subjects must be identified only by an identification code and not by their names. A third party (Virtual Surveys) will keep a separate confidential enrolment log that matches identifying codes with the subject's names and addresses.

2.2 Protocol Amendment(s)

Any modification to the agreed protocol must be approved in writing by the sponsor/ study co-ordinator and the investigator(s). If alterations have to be made to the protocol after Ethical approval has been given, then the amendments will be submitted for their approval and not implemented until approval is received.

2.3 Case Report Form

N/A

2.4 Source Document Verification

N/A

2.5 Adverse Event

In the current study no treatment or direct intervention is offered and no medical tests are performed. Therefore, it is considered unlikely that subjects will experience adverse event as a result of participation in the study. However, in the unlikely event that such an event occurs, feedback messages in the website have been designed in such a way as to minimise potential excess worry of subjects. Also, subjects presented with the Heart-Age with one highly elevated risk factor or high absolute CVD risk level (30%) can print and take a letter to their GP. This letter will present the GP with information on the study and the Heart-Age tool (Appendix 3). If further information is required the GP will be provided with the name of one of the investigators for discussion.

2.5.1 Adverse Event Definition

Any untoward medical occurrence in a study subject or administered a treatment and which does not necessarily have to have a causal relationship with the treatment. An adverse event can therefore be any unfavourable and unintended sign (including an abnormal laboratory finding), symptom, or disease temporally associated with the use of a treatment, whether or not considered related to the treatment. Pre-existing conditions that worsen during a study are to be reported as adverse events. They can become Serious Adverse Events if they fulfil one of the seriousness criteria described below.

SERIOUS ADVERSE EVENTS

Any adverse event that fulfils at least one of the following criteria:

- It is Fatal (results in death) (***note:*** death is an outcome, not an event).

- It is Life-Threatening (***note****:* the term "life-threatening" refers to an event in which the subject was at risk of death at the time of the event; it does not refer to an event which could hypothetically have caused death had it been more severe).
- It requires subject hospitalisation or prolongation of existing hospitalisation (***note***: "inpatient hospitalisation" refers to an unplanned, overnight hospitalisation).
- It results in persistent or significant disability/incapacity.
- It is a congenital abnormality/birth defect.
- It is medically significant or requires intervention to prevent one or other of the outcomes listed above.

Any adverse event that is considered SERIOUS must be reported. Since no treatment is offered to subjects and no medical tests conducted there is no such occurrence expected. Please see section 2.5.1

2.5.2 Unexpected Adverse Treatment Reaction

An adverse reaction, the nature or severity of which is not consistent with the expected risks of participating in the study.

2.5.3 Causality

Causality can be one of three possibilities:
- "NO" (definitely not treatment related)
- "YES" (remotely, possibly, probably or definitely treatment-related)
- "UNKNOWN"

All adverse events judged by either the investigator or the sponsor as being definitely not "NON TREATMENT-RELATED" qualify as Adverse Treatment Reactions.

2.5.4 Adverse Event Severity - Definition

The severity/intensity of adverse events can be graded either on a three-point W.H.O. scale (*See W.H.O. Handbook for Reporting Results of Cancer Treatment)* e.g.

- **Mild** or *Grade 1*: discomfort noted, but no disruption to normal daily activities.

- **Moderate** or *Grade 2*: discomfort sufficient to reduce or affect normal daily activities.

- **Severe** or *Grade 3*: Inability to work or perform normal daily activities.

2.5.5 Treatment and Follow-up of Adverse Events

All adverse events during the study will be documented and followed up by subject's GP who will be able to involve one of the study's investigators (Appendix 3) until the event is either resolved or adequately explained, even after the subject has completed his/her study.

2.6 Publication

Information relating to the study will be kept confidential and publications will be in a form with which the panellist cannot be identified.

2.7 Archiving

It is the responsibility of the investigator(s) to maintain adequate clinical study records. Copies of all clinical study material must be archived in the computer electronically (no hard-copies will be kept since this is an online study) for a minimum of five years from completion of the study (or more as legally required). All paper and electronic documents must be archived in a secure place and treated as confidential material.

2.8 Audit

The investigator agrees to comply with the sponsor and regulatory authority requirements regarding the auditing of the study.

3. Sponsor Responsibilities

3.1 General Responsibilities

The project manager/study co-ordinator, the investigator(s) and the study statistician will provide a final draft **protocol**. After mutual agreement, all parties sign the study protocol and submit the protocol to Ethics Committee.

The sponsor/study co-ordinator provides the investigator(s) with **sufficient material** and support to permit the investigator(s) to conduct the study according to the agreed protocol. The sponsor/study co-ordinator reserves the rights to **terminate the study prematurely** for persistent protocol violations, or any other valid and ethical reasons. Should this be the case, the necessary procedures will be arranged after review and consultation by both parties to ensure protection of the subject's interests.

The sponsor provides treatment and/or **compensation** in the case of an injury to, or the death of, a subject, as a result of his/her participation in a study sponsored by them. The sponsor also **insures** the investigator(s) against, or indemnifies them for, losses resulting from liability for such injuries or deaths.

3.2 Monitoring and Contract Research Organisation Responsibilities

N/A

3.3 Quality Assurance and Quality Control

All material used in studies are subjected to **quality control**. Quality assurance audits may be performed by the sponsor or any health authority during the course of the study or at study completion.

3.4 Archives

The protocol, approvals and all other essential documents related to the study must be archived, including certificates that satisfactory audit and inspection procedures have been carried out. All documents must be archived in a secure place for 5 years and treated as confidential material.

3.5 Final report

A summary will be issued within four weeks of study completion and a final report within 4 weeks of sponsor's comments. This report should include number of subjects enrolled, number of subjects dropped/withdrawn, adverse events, deviations, amendments, statistical analysis and general or specific comments from the investigator. Additionally, the contract laboratory will provide the sponsor/study co-ordinator with the electronic data.

3.6 Insurance

In the unlikely event of any subject suffering any significant deterioration in health or wellbeing arising as a result of taking part in this study, Unilever has undertaken to compensate them without having to prove that anyone involved in this study has been negligent. Any dispute will be referred to an arbitrator.

PART IV: Appendices

APPENDIX 1 - Study Schedule

APPENDIX 2 - Example of Informed Consent Form

APPENDIX 3 - Example of GP Letter

APPENDIX 4 – Questionnaires

APPENDIX 5 – Planning

APPENDIX 6– Recruitment Agency Screening

APPENDIX 7- HAPA Model Diagram

APPENDIX 8 – Participant Debrief

APPENDIX 1 - Study Schedule

**Phases**	**Processes**
**Phase 1:**<br><br>Recruitment/Screening<br>(Virtual Surveys)<br><br><br>Pre-Study Assessments	Screening questionnaire (height, weight, health status, age, gender, SES)<br><br>Online signing of consent form<br><br>Eligible subjects directed to the main Unilever study site to fill out a number of questionnaires
**Duration**	30mins
**Phase 2**<br><br>Conduction of main study	Depending on assigned condition:<br><br>-Completion of assessment to enable Heart-Age risk score computation and feedback provided by Heart-Age site<br><br>-Questionnaire assessing subject's reactions, perceptions, understanding of the tool and motivation to make lifestyle changes etc<br><br>-Subjects being asked to plan how to reduce their saturated fat intake over the next two weeks
**Duration**	Up to 30mins (depending on assigned condition)
**Phase 3**<br><br>Post-Study Assessments<br><br><br>Payment of participants	-Follow-up questionnaire assessing subject's reactions, perceptions, self-efficacy, planning behaviour, motivation to make lifestyle changes etc<br><br>-Subjects directed back to Virtual Surveys to be reimbursed and have their name entered into a prize draw
**Duration**	30mins

APPENDIX 2 - Example of Informed Consent Form

Study title: Heart-health and Saturated Fat Intake

You are being invited to take part in a research study. Before you decide it is important for you to understand why the research is being done and what it will involve. Take time to decide whether or not you wish to take part.

What is the purpose of the study?

The aim of this study is to assess your response to information we will give you about the health of your heart and saturated fat intake. This assessment is not a diagnostic and does not replace the advice of your GP.

Do I have to take part?

It is up to you to decide whether or not to take part. If you do decide to take part you will be asked to sign an online consent form. If you decide to take part you are still free to withdraw **at any time** and without giving a reason. You will be getting reimbursed at the end of Time 1 and again at the end of Time 3. If you withdraw at any time before the completion of a study phase then you won't get reimbursed for it.

What will happen to me if I take part?

You will be asked a number of health-related questions some of which you might have answered before. You will be provided with information on your heart-health and given the opportunity to provide your own feedback on your interaction with the website. You might also be asked to try and change aspects of your behaviour over the next few weeks. Once you have been through the consent form, there are three study phases: a) the first one will take approximately 30mins, b) the second one no more than an hour and c) the third one 30mins.

What are the possible disadvantages and risks of taking part?

You might realise that your heart-health and saturated fat intake is not as good as you were expecting. You might be also offered advice or a letter to visit your GP. This does not necessarily mean that you are unwell or that something is going to happen to you in the near future.

What are the possible benefits of taking part?

The most likely benefit is that you will learn something new about your health status and specifically your heart-health and saturated fat intake and receive recommendations on possible ways to improve it.

Will my taking part in this study be kept confidential?

All responses to this survey will be kept confidential. To protect your privacy and the security of the information you provide to the researchers your name or identity will not be collected or linked in any way to the research data.

Expenses and Remuneration

Upon completion of the study you will be reimbursed with £15 by the recruitment agency and will be entered into a prize draw of £200 in Amazon Vouchers. Please make sure that you click the appropriate button at the end of each phase to take you back to Virtual Surveys.

Who has reviewed the study?

This study has been reviewed by Colworth Research Ethics Committee.

Thank you for considering whether you want to take part in this study

By clicking the words I **AGREE** in the space below and by completing the attached survey I am giving my consent to participate in this study.

1. I confirm that I have read and understand the information for the above study.	Agree/Disagree
2. I understand that my participation is voluntary and that I am free to withdraw at any time, without giving any reason, without my medical care or legal rights being affected.	Agree/Disagree
3. I agree to take part in the above study.	Agree/Disagree

Agree with all of the above

APPENDIX 3 - Example of GP Letter

Date:
___/_____/200__
DD/MMM/YYYY

GP REFERRAL LETTER FOR STUDY SUBJECTS

Re: Patient's Name ______________________

DOB ___/_____/_____ (DD/MM/YY)

This letter is to inform you that the above-named patient took part in an internet-based health screening study for cardiovascular disease risk using algorithms based on data from the Framingham Heart Study. The study aims to help individuals understand their own level of risk in order to change their current lifestyle.

As part of this study the patient will have been asked for their total cholesterol level, their HDL cholesterol level, systolic blood pressure, whether they were being treated for high blood pressure or diabetic status, in addition to anthropometric measures such as age, gender, weight, height, waist circumference and lifestyle measures e.g. smoking status.

Subsequently individuals are given feedback based on a new "cardiovascular risk adjusted age" i.e.how appropriate their level of CVD risk is for their age – "Heart Age" – (see www.floraloveyourheart.ie for details). They are also given feedback on their individual risk factors, some of which may be unknown to them e.g. cholesterol level

Whilst knowledge of cholesterol and blood pressure is recommended by international guidelines, we do not wish to overburden doctors with requests for more cholesterol testing than is necessary. Therefore we have tailored our advice such that we recommend further testing (e.g. cholesterol test) for those only at potentially high CVD risk (>15% risk in the next ten years). The patient may therefore be asking you whether they need a cholesterol test or blood pressure measurement due to feedback that they may have higher CVD risk or high cholesterol (due to their age, BMI, presence of other risk factors). Of course this is not intended to replace clinical judgement and hope that this has served to facilitate a discussion of CVD risk with your patient

Please contact me on the above number if you would like to discuss this or require further information.

Yours sincerely,

Name Bob Hurling
Position Investigator

APPENDIX 4 – Questionnaires

Heart Age/CVD Risk Assessment Questionnaire

How old are you?

[ ]

Are you...

○ female ○ male

How tall are you?

[ ] centimetres

or

[ ] feet [ ] inches

How much do you weigh?

[ ] kilograms

[ ] Stones [ ] pounds

What is your waist measurement?

[ ] ◉ centimetres ○ inches

*Make sure the tape measure goes around your naval

Your general health

Do you have diabetes?

○ Yes ○ No

Has a doctor ever told you that you have had heart disease, or have you ever had a heart attack or heart surgery?

○ Yes ○ No

Do you currently smoke?

○ Yes ○ No

Have you ever been a smoker in the past?

○ **Yes** ○ **No**

How long ago did you stop smoking?

[ ] **years** [ ] **months**

Your cholesterol & blood pressure

It's a good idea to get your doctor to test your levels to check they're in the healthy ranges. Remember you can come back and do the test again, when you know.

My cholesterol level is

[ ] **mmol / L or**

[ ] **mg/dl** ☐ **I don't know**

My systolic* blood pressure is

[ ] **mmHg** ☐ **I don't know**

***Systolic blood pressure is the higher number of the two e.g. 140/90, 140 is the systolic blood pressure**

Have you taken, or are you currently taking, medication to lower your blood pressure?

○ **Yes** ○

Your family history

Your parents' medical history can give us clues about your health which will help calculate your estimated Heart Age.

Has your mother ever had heart disease? (This includes a heart attack, stroke or being diagnosed with heart disease by a doctor)

○ **Yes** ○ **No** ○ **I don't know**

How old was your mother when she was diagnosed or had a heart attack?

[ ] **Years**

Has your father ever had heart disease? (This includes a heart attack, stroke or being diagnosed with heart disease by a doctor)

○ **Yes** ○ **No** ○ **I don't know**

How old was your father when he was diagnosed or had a heart attack?

[ ] **Years**

What you eat

How often do you eat at least 5 servings of fruit and/or vegetables each day?

- 0-1 times a week
- 2-4 times a week
- 5 or more times a week

How often do you choose foods that are lower in saturated and trans fat?

- 0-1 times a week
- 2-4 times a week
- 5 or more times a week

(Examples of foods that are high in saturated fat are: red meat and dairy produce. Examples of foods that are high in trans fat are: fried foods and some convenience products)

How often do you choose foods that are lower in salt?

- 0-1 times a week
- 2-4 times a week
- 5 or more times a week

How often do you eat fast food or ready-made meals (not including low calorie versions)?

- 0-1 times a week
- 2-4 times a week
- 5 or more times a week

How often do you try to limit your daily calorie intake?

- 0-1 times a week
- 2-4 times a week
- 5 or more times a week

Exercise & lifestyle

We consider exercise to be things like walking and working out at the gym for a period of more than 15 minutes.

Over the past week, how often did you perform any of the following activities for at least 15 minutes on each occasion? Brisk walking, jogging, running, cycling, swimming, gym, or any other vigorous exercise.

- 0-1 times a week
- 2-4 times a week
- 5 or more times a week

How active is your everyday life (either at work or at home)?

- Mainly sitting
- On feet
- On feet and using tools/lifting objects
- Very

SOCIAL COGNITIVE DETERMINANTS AND FOOD FREQUENCY QUESTIONNAIRES

Questionnaire Instructions -Time 1

Dear Participant,

Thank you for taking part in this study, which is divided in three stages. In this first stage, you will be asked a series of questions about your dietary knowledge, eating habits and beliefs.

Once you have completed all three phases of the study you will receive a reward of £15. If you have any difficulties completing this form, please contact ____________ in Virtual Surveys.

This first stage should not take longer than 30mins. Please take time to carefully read and answer each question. Try to complete each stage in one session.

Questionnaire Instructions -Time 2

Dear Participant,

Welcome back to this study looking at eating beliefs and habits, specifically in relation to saturated fats.
This stage should not take longer than 30 minutes and you will be asked again about your dietary beliefs.

We all need fat but choosing the right type of fat can influence the health of our hearts. You can reduce your chances of developing heart disease by eating a diet that is high in fruit and vegetables, high in fibre, and low in salt and fat. Although it's important to try to eat less fat, we also need to think about the types of fat we are eating. We should be cutting down on food that is high in saturated fat or trans fats or replacing these foods with ones that are high in unsaturated fat instead.

Unsaturated fats can be a healthy choice. These types of fats, include foods such as oily fish, avocados, nuts and seeds, sunflower, olive oil and spreads- and can actually reduce cholesterol levels and provide us with the essential fatty acids that the body needs.

Saturated fats come from animals and certain plant oils. According to the Foods Standard Agency, a food that has more than 5g saturated fat per 100g is classified as being high in saturated fat.
http://www.eatwell.gov.uk/healthydiet/nutritionessentials/fatssugarssalt/fats

Foods high in saturated fat

Eat these sorts of foods less often or in small amounts:

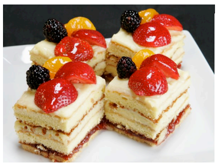
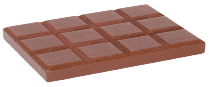
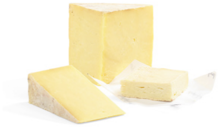

Red Meat, pies, sausages
Ice-Cream
Hard Cheese
Chocolate
Coconut Oil, coconut cream

Cakes & Biscuit
Full-fat -Milk
Pastries
Butter & Lard

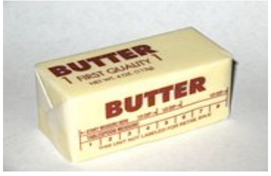

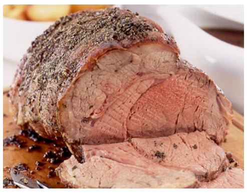

Please take time to carefully read and answer each question. In two weeks, you will be contacted again by email and asked to complete a brief follow-up questionnaire online.

Questionnaire Instructions -Time 3

Dear Participant,

Thank you very much for taking part in this study.This is the final and most important stage in which you will complete the Eating Habits and Dietary Beliefs questionnaires again.

Upon completion of this stage you will be directed back to Virtual Surveys to receive your incentives and your name will be entered into a Prize Draw of Amazon Vouchers.

This stage should not take longer than 30 minutes!

SATURATED FAT INTAKE QUESTIONNAIRE

Please choose the frequency with which you have eaten each of the foods in the list below.

**Food**	2 or more times per day	Every day	Three to five times per week	One to two times per week	One to three times per month	Rarely or never
Whole milk						
Semi-skimmed milk						
Skimmed milk						
Butter						
Margarine						
Polyunsaturated margarine						
Low fat spread						
Ice cream						
Yoghurt						
Cheese- ordinary						
Cheese- low fat						
Eggs- fried						
Eggs- not fried						
Cheese/egg meals						
Beef- roast/steak						
Lamb- roast/chops						
Pork- roast/chops						
Chicken/Turkey						
Bacon						
Meat dishes						
Canned meats						
Meat pies/pasties						
Sausages/beefburgers						
Liver/Kidney/Pate						
Fish- not fried						
Fish-fried						
Fish-canned						
Bread-white						
Bread-brown/granary						
Bread-wholemeal						
Sweet biscuits						
Crackers/crispbread						
Cakes/buns/pastries						
Puddings						
Breakfast Cereal-High fibre						
Breakfast Cereal-Ordinary						
Breakfast Cereal-Muesli						
Rice/Pasta						
Apples/Pears						
Oranges/Grapefruits						
Bananas						
Green Vegetables						
Carrots/Tomatoes						

Other Vegetables						
Baked beans						

**Food**	2 or more times per day	Every day	Three to five times per week	One to two times per week	One to three times per month	Rarely or never
Other beans/lentils						
Vegetable dishes						
Potatoes- fried						
Potatoes- not fried						
Beer/lager						
Wine/sherry/spirits						
Tea/Coffee						
Squash/Fizzy drinks						
Low calorie drinks						
Pure fruit drinks						
Chocolate						
Sweets						
Sweet spreads						
Sugar						
Crisps and other savoury snacks						
Nuts (including peanut butter)						
Sauces and pickles						
Salad oil/ dressing/mayonnaise						

	Strongly Disagree					Strongly Agree	
I have eaten foods low in saturated fat in the last week	1	2	3	4	5	6	7

	Never				Frequently		
How often did you eat foods low in saturated fat in the last week?	1	2	3	4	5	6	7

Intentions

Please rate your intentions to change your saturated fat intake over the next two weeks.

Over the next two weeks I intend to….

	Strongly Disagree 1	2	3	4	5	6	Strongly Agree 7
Reduce the amount of fat/oil when cooking							
Eat smaller portions of high fat foods							
Replace high fat with lower fat alternatives							
Use margarine rather than butter when cooking							
Choose lower fat foods when eating out							

Eat take-away foods less often							
Increase the amount of vegetables I eat							
Increase the amount of fruit I eat							
Use margarine rather than butter when spreading							
Reduce the amount of saturated fat in my diet							

Expected Outcomes

**What do you think would happen if you reduce your saturated fat intake?**<br><br>**If I reduce my saturated fat intake….**				
	Not at all true<br>1	Barely true<br>2	Mostly true<br>3	Exactly true<br>4
I will feel good				
That will reduce my risk of heart-disease				
I will be able to eat more foods without getting too many calories				
I will feel physically more attractive				
I won't have weight problems anymore				
Food won't taste as good				
That will impair my social life (e.g. at parties with friends)				
That will be good for my blood pressure				
I will have to make an effort of buying the right products				
That will be good for my cholesterol				
I will have to spend more time on preparing meals				

Confidence in Saturated Fat Reductions

Various barriers could make it difficult to reduce your saturated fat intake. How certain are you that you could reduce it?

I am confident I could reduce my saturated fat intake even if…				
	Not at all<br><br>1	Barely true<br>2	Mostly true<br>3	Exactly true<br><br>4
I will have to make a detailed plan describing how to remember to buy low saturated fat foods				
I have to rethink my behaviours and opinions concerning low saturated fat foods				
I will have to overcome my different high fat habits				
I will have to push myself				

Maintaining Saturated Fat Reductions

How certain are you that you could overcome difficulties when trying to maintain reductions in your saturated fat intake?

I am certain that I could overcome difficulties related to maintaining reductions in my saturated fat intake even if….				
	Not at all<br>1	Barely true<br>2	Mostly true<br>3	Exactly true<br>4
I am eating out				
High fat foods are more widely available than low fat foods				
I am tired				
I am stressed out				
I feel tense				
I don't see success at once				
My cholesterol does not improve immediately				
I wont get support for my first attempts				
It takes long to make it a habit				
My partner/family/friends are not trying to reduce their saturated fat intake				
I cannot see any positive changes immediately				

Risk Perceptions

Please read the following statements and choose the one that best corresponds to you.

My chances of getting heart-disease in the short-term (e.g. next couple of years) are								
Not at all strong	1	2	3	4	5	6	7	Very strong

Compared to an average person of my age and sex, my chances of getting heart-disaese are						
Much below average<br>1	Below average<br>2	A little below average<br>3	Average<br>4	A little above average<br>5	Above Average<br>6	Much more above average<br>7

Saturated Fat Intake Plans

I now have my own plan regarding				
	Not at all	Barely true	Mostly true	Exactly true
When to reduce my saturated fat intake				
How to reduce my saturated fat intake				

Feedback on the Information Received

I felt that the information provided by the study on heart-disease and saturated fat intake was:							
	Strongly Disagree					Strongly Agree	
Trustworthy	1	2	3	4	5	6	7
Of personal relevance to me							
Enjoyable							
Interesting							
Contained a lot of new information							
Credible							
Worrying							
A wake up call							

APPENDIX 5- Instructions on Planning

Planning

We would like you to plan how to reduce your saturated fat intake over the next two weeks. Below is a list of situations, e.g. I'm in the shops, and a list of different behaviours, e.g. I will choose margarine. Identify **as many situations** in which you would like to change your saturated fat intake and then try to match it with behaviours from the list below.

Choose behaviours that you would like to change rather than ones that you are already doing. By choosing a situation in which you are tempted to eat high saturated fat foods and then ways to overcome the temptation you are more likely to successfully reduce your saturated fat intake!

For example, you might find it useful to plan to put margarine on your bread instead of butter at lunch time. Then the situation-behaviour statement will become

e.g.

If I am ………having lunch……**Then** I will………choose Margarine (on bread).

Situations		Behaviours
**If**……………		**Then**……………
I'm in the shop		I will choose a lower fat dairy product or spread
I'm having lunch		I will go for fruit
I'm getting a snack		I will go for vegetables or salad
I'm having dinner		I will check it's 5g or less in saturated fat per 100g
I'm having breakfast		I will find out and choose the lower fat option
I'm in a restaurant		I will go for grilled/steamed poultry or fish
I'm having a good time when I'm out with friends		I will go for a short walk
I am feeling depressed or down		I will call or find someone to talk to
I'm craving a high fat food		I will tell myself that if I try hard enough I can eat healthily
I'm feeling hungry		I will distract myself with something else
I had a hard day		I will reduce the amount of food I eat
Others around me are eating high-fat foods		I will not buy high fat foods to keep in the house
I'm having a coffee/tea break		I will avoid others who are eating high fat foods

APPENDIX 6 – Recruitment Agency Screening

Health Check Survey: Draft Questionnaire v2 2010 – Email Invite

Invite :

From Chris Robins
Subject: Help us with our research

Dear panel member,

Today, we are inviting you to take part in a Health Check survey focusing on heart-health and healthy eating, which will involve carrying out a number of tasks. This will involve interacting with the system at three different times. The first time will not take more than 30 minutes. All those eligible and completing all three different interactions will be paid <<pointsAsMoney>> and their name will be also entered into a prize draw.

Instructions on how to take part can be found below. We look forward to hearing your opinions.

You can participate with this research project until <<end_date>>. Please note this research project is limited to 900 respondents. Once 900 suitable respondents have completed the survey the project will be closed.

Chris Robins
SSI

Health Check Survey: Draft Questionnaire v1 2010

Page 1: Welcome

Intro

Thank you for your interest in this survey. Please can you answer the following profiling questions by clicking on a box for each. This profiling survey is being conducted by an independent market research agency in accordance with the Market Research Society Code of Conduct. More info on survey?

For more information about the survey please click here.

Age

[Ordered/Single]

Q1 What year were you born?

Answers

- Show as drop down list for the past 80 years (1927 to – last code – after 1991)
- List with most recent at the top

SCREEN OUT IF BORN FROM 1991 ONWARDS

Create a hidden variable which means that age is grouped into the following bands for quotas/monitoring:

29 or under	1	CLOSE
30 to 45 years	2	
46 to 60 years	3	
61 or over	4	CLOSE

MaleFemale

[Ordered/Single]

Q2 Are you male or female?

Answers

Male	1
Female	2

Region

[Ordered/Single]

Q3 Where do you live?

Answers

London/South East	1	
South West	2	
Eastern	3	
Midlands	4	
Wales	5	
Yorkshire & The Humber	6	
North West	7	
North East	8	
Scotland	9	
Northern Ireland	10	
Outside UK	11	CLOSE

Survey Notes

[Info]

Important notes:

1. This is a genuine market research survey and not selling you anything

2. We make privacy a top priority - read our policy at http://www.virtualsurveys.com/privacy/
3. Whilst completing this survey your normal Internet connection charges will apply
4. With this survey, your computer has been sent a small piece of information (a cookie) to help us administer the survey. We also collect system data which will not be attributed to you personally
5. If you have any queries about these notes, or the survey, please email andy.smith@virtualsurveys.com or ring +44 (0)161 242 1100
6. If you do not wish to complete this survey, simply close this window
7. Virtual Surveys is a UK based company and complies with UK and EU Data Protection Laws, but you should be aware that the Internet is an international medium and this may result in your data being processed outside the EU, you should only proceed with this survey if you consent to this

Page 2: About You

PersonalInfo
[Info]
Note this information will be used for analysis purposes only and will not be attributed to you personally

Height
[Ordered/Multi]
Q4 How tall are you?
Answers

Centimetres	1	OPEN
Feet	2	OPEN
Inches	3	OPEN
Don't Know	99	Excl

Weight
[Ordered/Multi]
Q5 How much do you weigh?
Answers

Killograms	1	OPEN
Stones	2	OPEN
Pounds	3	OPEN
Don't Know	99	Excl.

Q6 **BMI (HIDDEN QUESTION)**
[Ordered/Single]
Answers

29.9	6	Excl
30	7	
31	8	
32	9	
33	10	
34	11	
35	12	
36-40	13	
40+	14	

Smoker
[Ordered/Multi]
Q7 Which of these phrases best describes your smoking habits?
Answers

Smoker	1	
Non smoker	3	

Heart
[Random/Ordered/Single]
Q8 Which, if any, of the following health problems have you ever been diagnosed with?
Answers

Diabetes	1	
Heart disease	2	CLOSE
High blood pressure	3	
Cancer	4	CLOSE
Any other chronic disease of the major organs (e.g kidney failure etc.)	5	CLOSE

Income
[Ordered/Single/ two columns]
Q9 Please indicate which of these income bands come closest to your total gross annual income before tax and other deductions?
Answers
1. Less than £8,000
2. £8,000 to £12,999
3. £13,000 to £22,999
4. £23,000 to £33,999
5. £34,000 to £49,999
6. £50,000 to £66,999
7. £67,000 to £99,999
8. £100,000 to £134,999
9. £135,000+
10. Don't know
11. Prefer not to answer

SES
[Ordered/Single/ two columns]
Q 10 Which of the categories below best describes your current employment?
Answers
1. Homemaker/ housewife
2. Retired
3. Unemployed/ on benefit
4. Factory/ manual/ unskilled worker
5. Crafts/ tradesperson/ skilled worker
6. Office/ clerical/ administration
7. Middle management
8. Senior management
9. Professional
10. Student/ full time education
11. Don't know

SCREENED:

- AGE: Under 30 and over 60 (Q1/1 or 4)
- IF BMI <30
- DIAGNOSED WITH HEART DISEASE, CANCER OR ANY OTHER CHRONIC DISEASE OF THE MAJOR ORGANS AT Q8 (CODE 2, 4 OR 5)
- OUT OF QUOTA

Redirect: SSI thank you page (no payment)

QUOTAS (PER COUNTRY) / ASSIGN TO CELL:

HA+Planning		Heart-Age		Planning		Control Group	
Age	Gender	Age	Gender	Age	Gender	Age	Gender
30-45 years (N=50)	*Males (25)*	*30-45 years (50)*	*Males (25)*	*30-45 years (50)*	*Males (25)*	*30-45 years (50)*	*Males (25)*
	Females (25)		*Females (25)*		*Females (25)*		*Females (25)*
45-60 years (N=50)	*Males (25)*	*45-60 years (50)*	*Males (25)*	*45-60 years (50)*	*Males (25)*	*45-60 years (50)*	*Males (25)*
	Females (25)		*Females (25)*		*Females (25)*		*Females (25)*

ELIGIBLE RESPONDENTS

Page 3 – Next Steps

Thank you for helping with this survey. Now please click the 'Complete Survey' button below to close this profiling survey. You will then be taken to the Health Check Website which will tell you more about the research process.

qcells
[Single/Hidden]
Switch1 Alternatively directed to one of two cells?
Answers

Cell 1 (Unilever to provide URL)	1
Cell 2 (Unilever to provide URL)	2

Process thereafter

1. **Respondents passed to Health Check Survey – Stage 1**
2. **Respondents passed to Feedback Survey – Stage 2**
3. **Respondents passed back to VSL Survey – Stage 3**

Page 4 – Health Check Survey

Other Comments
[Open]
Q9 Do you have any other comments on the tasks carried out or the survey you have just completed?
Please type your comments in the space provided.
