# Supplementary material for: Web-Based Risk Communication and Planning in an Obese Population: Exploratory Study
Source: J Med Internet Res. 2011 Nov 24;13(4):e100. doi: 10.2196/jmir.1579 (PMC3278086; doi:10.2196/jmir.1579)
Supplement: Supplementary file 2 [file jmir_v13i4e100_app2.pdf]

Unilever

Unilever Colworth
Colworth Park
Sharnbrook
Bedford
MK44 1LQ
United Kingdom

T: +44 (0)1234
F: +44 (0)1234

From: Kathey Towler
On behalf of the CREC

To: Natasha Soureti

Date: 4th December 2008

Ethical Approval

Study Reference: Heart-Age and Planning in the Promotion of Saturated Fat Intake Reductions

Signed for the Committee............ K Towler ....

Name............ K Towler ....

INVESTOR IN PEOPLE

Unilever UK Central Resources Limited
Registered number 29140
Registered office Unilever House,
Blackfriars, London EC4P 4BQ
